# Evaluation of the efficacy of systemic therapy for advanced uterine leiomyosarcoma: A systematic review, meta‐analysis, and meta‐regression analysis

**DOI:** 10.1002/cam4.5930

**Published:** 2023-04-20

**Authors:** Iqra Ijaz, Muhammad Naveed Shahzad, Hossein Hosseinifard, Shuya Liu, Masoud Ostadi Sefidan, Lubna Ejaz Kahloon, Saber Imani, Zhong Hua, Yu Qin Zhang

**Affiliations:** ^1^ Sichuan Provincial Center for Gynecological and Breast Diseases Southwest Medical University Luzhou Sichuan PR China; ^2^ Department of Obstetrics & Gynecology, Holy Family Hospital Rawalpindi Medical University Rawalpindi Pakistan; ^3^ Stem Cell Laboratory, Department of Hematology The Affiliated Hospital of Southwest Medical University Luzhou Sichuan PR China; ^4^ Department of Biostatistics, School of Public Health Hamadan University of Medical Sciences Hamadan Iran; ^5^ Department of Oncology The Affiliated Hospital of Southwest Medical University Luzhou Sichuan PR China; ^6^ Department of General Surgery, Rasool‐e Akram Hospital Iran University of Medical Sciences Tehran Iran; ^7^ Shulan International Medical College Zhejiang Shuren University Zhejiang Hangzhou PR China; ^8^ Department of Obstetrics The Affiliated Hospital of Southwest Medical University Luzhou Sichuan PR China

**Keywords:** meta‐analysis, meta‐regression analysis, systemic therapy, uterine leiomyosarcoma

## Abstract

Uterine leiomyosarcoma (uLMS) is an aggressive mesenchymal neoplasm associated with a poor prognosis. Systemic chemotherapy is the standard therapy for patients with uLMS. However, it is unclear which treatment regimen results in the most favorable clinical outcome. We performed a meta‐analysis and meta‐regression analysis to assess the efficiency of different treatments received by patients with advanced, metastatic, and relapsing uLMS by evaluating the objective response rate (ORR) and disease control rate (DCR) as primary endpoints. The frequentist random effects meta‐analysis model was used to compare the outcomes of different treatment regimens for advanced uLMS. A meta‐regression analysis was performed to estimate the association between the study‐specific hazard ratios and specific demographic variables. A meta‐analysis of 51 reports including 1664 patients was conducted. Among patients who received adjuvant chemotherapy (916 patients; 55%), gemcitabine and docetaxel were the most frequently used drugs. First‐line monotherapy with alkylating agents (pooled ORR = 0.48; 95% confidence interval [CI]: 0.44–0.52) and second‐line monotherapy with protein kinase inhibitors (pooled ORR = 0.45; 95% CI: 0.39–0.52) resulted in favorable prognoses. The combinations of anthracycline plus alkylating therapy (pooled DCR = 0.74; 95% CI: 0.67–0.79) and of gemcitabine plus docetaxel (pooled DCR = 0.70; 95% CI: 0.63–0.75) showed the greatest benefits when used as first‐line and second‐line chemotherapies, respectively. Subgroup meta‐analysis results revealed that dual‐regimen therapies comprising anthracycline plus alkylating therapy and gemcitabine plus docetaxel are practical therapeutic choices for International Federation of Gynecology and Obstetrics stages III–IVb with distant metastases when assessed by computed tomography (*p* = 0.001). Furthermore, neoadjuvant chemotherapy and local radiotherapy resulted in favorable outcomes for patients with earlier stages of distant relapsed uLMS (*p* < 0.001). Our findings provide a basis for designing new therapeutic strategies and can potentially guide clinical practice toward better prognoses for uLMS patients with advanced, metastatic, and relapsing disease.

## Graphical abstract

Subgroup meta‐analysis results revealed that dual regimen therapy with anthracycline + alkylating agents and gemcitabine + docetaxel was an effective therapeutic choice when assessed by computed tomography methods in FIGO stage III–IVb with bone metastases (*p* 0.001). Our meta‐regression findings showed that patients with a history of neoadjuvant chemotherapy and local radiotherapy in earlier stages of the disease have an overall improvement in the outcome of recurrent uLMS receiving current therapy (*p* < 0.001). We could not observe a significant logistic regression correlation between the median age of patients and uLMS cancer risk (*p* > 0.05).

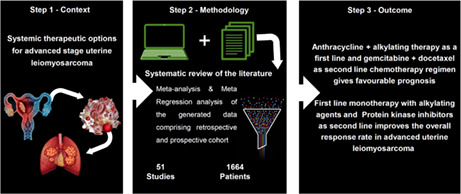

## 1 INTRODUCTION

Uterine leiomyosarcoma (uLMS) is the most frequently diagnosed subtype of uterine sarcoma, with an incidence of 0.8/100,000 cases annually.^1^ Furthermore, uLMS is the age‐related malignant form of uterine sarcoma that affects between 20% and 65% of women by 40 years of age.^1^ Hysterectomy is the standard treatment for uLMS if patients are diagnosed during the early stage of disease. Although many clinical risk factors are used to determine the early diagnosis and prognosis of patients with uLMS, these patients still have poor prognoses.^2^, ^3^ The 5‐year overall survival (OS) rate of uLMS is more than 50% for early International Federation of Gynecology and Obstetrics (FIGO) stages, and those for FIGO stages I and II are 75.8% and 60.1%, respectively.^4^ Conversely, stages III and IV of uLMS with refractory disease and high recurrence rates have 5‐year OS rates of 34.9% and 28.7%, respectively. Despite complete tumor resection and treatment, the relapse risk for patients with uLMS remains between 50% and 70%. Therefore, the National Comprehensive Cancer Network and FIGO uterine cervical cancer staging system recommend the following different therapeutic guidelines for patients with uLMS: observation for FIGO stage I; systemic therapy for FIGO stage II; and adjuvant chemotherapy for FIGO stages III and IV.^5^, ^6^

Non‐targeted alkylating agents such as Adriamycin, dacarbazine, trabectedin, pazopanib, eribulin, and vinorelbine for monotherapy or combination therapy are the most frequently studied treatments for FIGO stages II and III. Recently, many cohort studies have been designed to determine the prognostic value of molecular‐targeted and immune checkpoint therapy for uLMS patients with advanced, metastatic, and relapsing disease. In this regard, the combination of the monoclonal antibody olaratumab with adriamycin for uLMS patients with FIGO stages III and IV initially showed some benefits in relation to OS, although recent data have shown conflicting results.^7^ Furthermore, protein kinase inhibitors (PKIs) such as pazopanib and sorafenib were successfully used during randomized, phase III, clinical trials, but their clinical use has not been implemented.^6^ Wide ranges of objective response rates (ORRs) and disease control rates (DCRs) have been reported for non‐targeted alkylating therapy and anthracycline for an acceptable number of patients.^7^ In contrast, immunotherapy, such as pembrolizumab and lenvatinib, has been demonstrated to be impractical because some studies of uLMS have claimed low success rates.^8^ Two retrospective reviews reported low response rates or suggested that the response was not better than that of chemotherapy for the disease, although these observations were uncontrolled. The relapse rate after treatment is high, and continued treatment is usually uncontrollable, which may lead to an unacceptably high cumulative dose of pembrolizumab.

Accordingly, several researchers have attempted to find an effective modality to treat uLMS without significant side effects, especially for patients with metastatic uLMS. Undoubtedly, a comparison of the efficacy of different therapies for uLMS patients with advanced, metastatic, and relapsing disease could determine effective treatment strategies and increase the OS of patients with uLMS. Further population‐based studies are needed to compare the efficacy of different therapies.

This study aimed to conduct a comprehensive comparative meta‐analysis and meta‐regression analysis using a large sample of patients with uLMS to determine the most effective therapeutic options for those with advanced, metastatic, and relapsing uLMS. Specifically, this study aimed to compare the impact of different treatments on the ORR and DCR of uLMS patients and evaluate additional prognostic factors such as age, FIGO stage, and the effects of previous therapies on treatment outcomes. This study sought to provide important insights regarding the optimal treatment approaches for uLMS patients. Additionally, this study aimed to identify any potential gaps or inconsistencies in the current literature regarding the treatment of uLMS to inform future research and clinical practice. Furthermore, this study aimed to provide a robust and evidence‐based foundation for the development of personalized treatment plans for uLMS patients that may ultimately lead to improved patient outcomes and quality of life.

## 2 MATERIALS AND METHODS

### 2.1 Research strategy and study identification

This quantitative meta‐analysis was conducted by systematically following the recommendations of the Preferred Reporting Items for Systematic reviews and Meta‐Analyses statement guidelines. A literature search was conducted using the MEDLINE electronic databases PubMed, Embase, Wiley, ISI Web of Science, Science Direct, Google Scholar, and VIP database. All databases were searched without language and geographical restrictions. Two independent authors (I.I. and M.N.S.) analyzed all publications until November 15, 2022, to assess the effects of systemic therapies on advanced, metastatic, or relapsed unresectable uLMS. Medical subject heading terms used for the search were as follows: “therapy” or “chemotherapy” or “immunotherapy” or “targeted therapy” or “hormonal therapy” and “uterine sarcoma” or “uterine leiomyosarcoma” or “uLMS.” Only articles in English were considered. Relevant articles and their corresponding reference lists were also reviewed. All selected works were retrieved and screened by two separate investigators.

### 2.2 Inclusion and exclusion criteria

The inclusion criteria were as follows: studies published in English with a minimum sample size of five patients; uLMS samples confirmed by histopathological diagnostic analyses; therapeutic efficacy assessed by response evaluation criteria in solid tumors (RECIST) (these criteria comprise complete response, partial response, stable disease, progressive disease, ORR [complete response + partial response], and DCR [complete response + partial response + stable disease]); and randomized trials and observational studies including patients with advanced, recurrent, or progressive uLMS. The exclusion criteria were as follows: articles addressing the pediatric population and those dedicated exclusively to early‐stage disease, carcinosarcoma, and other uterine cancers; case reports, case series, review articles, editorial articles, early phase trials, and reports; non‐English articles; duplicate works or continued work of previous publications; unqualified key data such as insufficient data for treatment and *p* value calculation; and articles containing uLMS data with ambiguous mixed results and unspecified treatment regimens.

### 2.3 Data collection

The titles and abstracts of all articles selected based on the population, intervention, control, and outcomes principles were first analyzed by two independent investigators (I.I. and M.N.S.).^9^ Then, full article screening was performed, and the following key demographics and clinicopathologic parameters were recorded: the name of the first author; publication year; ethnicities of the patients; country where the studies were performed; patient sample size; study design; study phase; FIGO disease stage; endpoints according to RECIST; drugs used; and other summary estimates for a total of 30 variables in our database.^10^ We also contacted the authors of the selected articles to obtain any relevant additional information. Any discordance was settled through discussion; when a consensus was not reached, a third investigator (S.I.) was consulted to find a compromise. The study was supplemented by searching the reference lists to identify additional studies. When any of the aforementioned information was unavailable from the reports or authors, the item was marked as “not reported.” After all the aforementioned criteria were met, we focused on the study of uLMS, the most frequent subtype involved in the screened trials, and the subtype with good sample strength.

### 2.4 Quality assessment

The quality of the included studies was evaluated according to the Newcastle‐Ottawa scale (NOS),^11^ and the treatment accuracy of the studies was assessed by the Quality Assessment of Diagnostic Accuracy Studies 2 (QUADAS‐2) protocols.^12^ The QUADAS‐2 tool was used for patient selection, index testing, reference standardization, and flow timing. The total NOS score ranges from 0 to 9 points; a score of 6 points is the threshold for high‐quality classification. Low‐quality studies (scores ≤4 points) were excluded. Study bias was calculated according to the Cochrane Collaboration tool (Cochrane handbook for systematic reviews of interventions version 5.1.0). Briefly, using the Cochrane Collaboration tool, each assessment included seven questions to be answered with “yes,” “no,” or “unclear.” “Yes” answers indicated that the bias risk of the study was considered low, whereas “no” and “unclear” answers indicated that the risk of bias was considered high.

### 2.5 Meta‐regression analysis

A meta‐regression analysis was performed to assess which factors determine the observed heterogeneity between studies. Our goal was to clarify the influence of factors (median patient age, publication year, OS reported by each study, and effects of neoadjuvant chemotherapy [NACT; second‐line therapy] or local radiotherapy [first‐line therapy]) on the average difference between the ORR and DCR of patients with advanced uLMS undergoing current therapy. To further explore sources of study heterogeneity, we fitted meta‐regression models to estimate the association between the study‐specific ORRs and DCRs and ages of participants, primary endpoints (i.e., ORR, DCR, and mOS), timeframe for assessing weight changes, previous therapies (i.e., chemotherapy and radiotherapy), and proportion of the baseline sample included in the analysis.

### 2.6 Statistical analysis

A systematic search and data combination was performed using EndNote Software version X9. Generally, data are presented as the mean (±standard deviation) or median (range), but qualitative variables were described as numbers and percentages. Random and fixed effects models were used to determine the pooled estimates of the impact of different systemic regimens on the prognosis of advanced uLMS (in terms of the ORR and DCR as well their association with other factors associated with the prognostic value). The Cochrane *Q* statistic and Higgins *I* ^2^ statistic were applied to test the heterogeneity between studies. Statistical significance was considered *p* < 0.05 and/or *I* ^2^ > 0% by the fixed effects. Additionally, subgroup analyses were conducted using the research design, median patient age, number of pharmacological agents comprising combination therapy, line of therapy, disease stage, and publication year to ascertain the cause of heterogeneity between studies of the ORR and DCR. All statistical analyses were performed using R software (version 4) packages, including the “mada” package (The R Foundation). Begg's funnel plots and the Egger's linear regression test were used to evaluate publication bias.

## 3 RESULTS

### 3.1 Literature search and characteristics of selected studies

**FIGURE 1:**
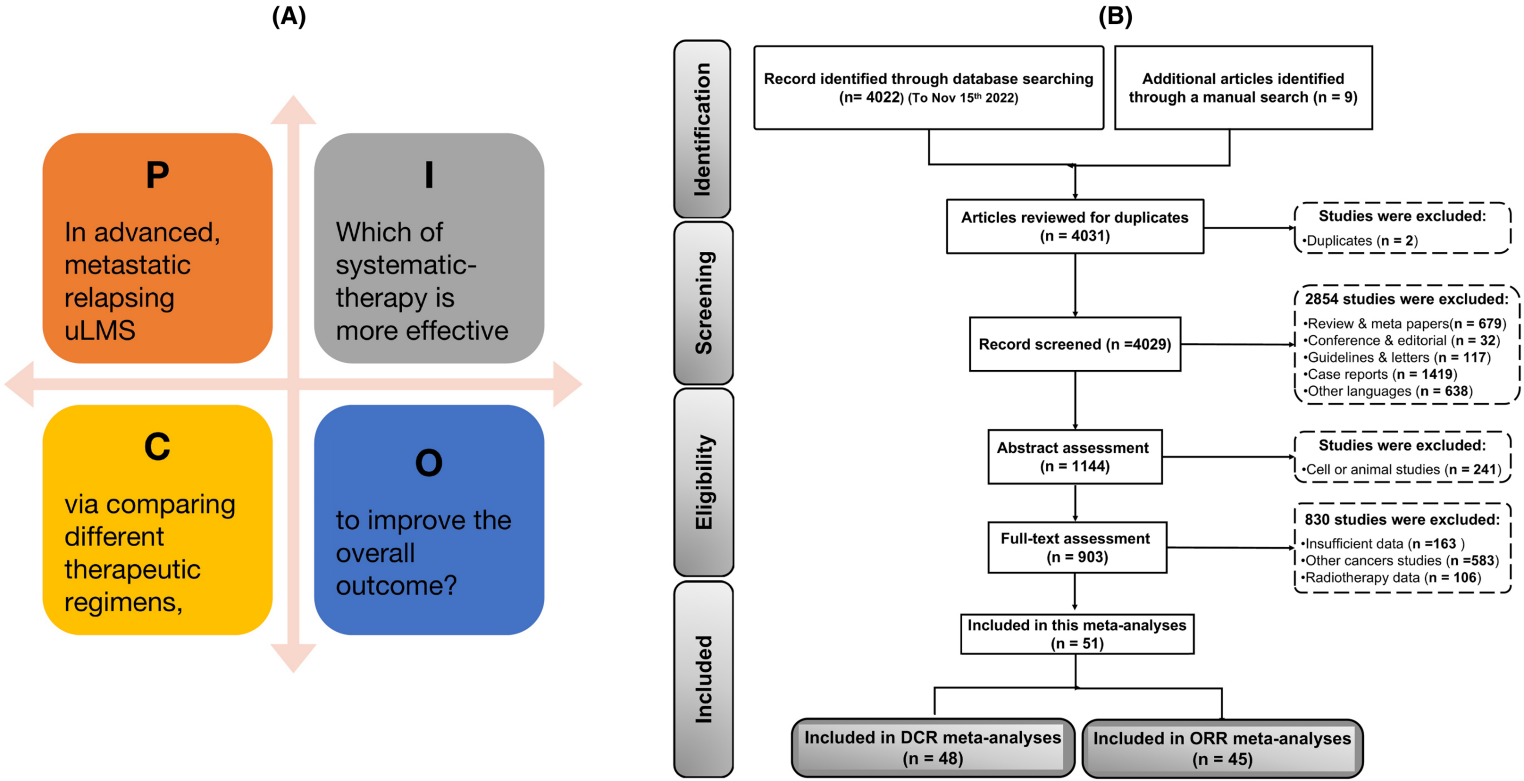
Flowchart of the rationale for study selection following Preferred Reporting Items for Systematic reviews and Meta‐Analyses [PRISMA] guidelines (*n* = number of studies).

The comprehensive systematic search strategy was established and used to define the clinical issues according to the population, intervention, control, and outcomes principle (Figure 1A). A detailed Preferred Reporting Items for Systematic reviews and Meta‐Analyses study flowchart of the identification, screening, and exclusion processes is shown in Figure 1B. For this meta‐analysis, 4031 studies were retrieved; of these, 4022 were retrieved from online databases and 9 were manually curated. Two reports were excluded because they were duplicate studies. After carefully reviewing the abstracts, 3126 studies were excluded because they were reviews or meta‐analysis reports (679 studies), conferences or editorials (32 studies), guidelines or letters (117 studies), case reports (1419 studies), articles written in languages other than English (638 studies), and cell or animal studies (241 studies). Of the remaining 903 full‐text candidate articles, potential studies were excluded because they included insufficient data (163 studies), cases of non‐uterine cancer (583 studies), and radiotherapy data (106 studies). The included and excluded full‐text articles are listed in Tables **S1** and S2. Finally, 51 studies were included in the current meta‐analysis and meta‐regression analysis. The data showed that 83% of patients had metastatic or advanced uLMS and 40% had relapsing uLMS. Of the 51 relevant studies describing 1664 cases, 48 studies were included in the DCR analysis and 45 studies were included in the ORR analysis (Figure 1B).^13^, ^14^, ^15^, ^16^, ^17^, ^18^, ^19^, ^20^, ^21^, ^22^, ^23^, ^24^, ^25^, ^26^, ^27^, ^28^, ^29^, ^30^, ^31^, ^32^, ^33^, ^34^, ^35^, ^36^, ^37^, ^38^, ^39^, ^40^, ^41^, ^42^, ^43^, ^44^, ^45^, ^46^, ^47^, ^48^, ^49^, ^50^, ^51^, ^52^, ^53^, ^54^, ^55^, ^56^, ^57^, ^58^, ^59^, ^60^, ^61^, ^62^

The potential studies included in this systematic analysis were published between 1979 and 2019. Demographic characteristics and RECIST results of systemic therapy for uLMS patients are summarized in Table 1. Clinicopathologic features assessed during the sub‐meta‐analysis and meta‐regression analysis are outlined in Table S3. Studies were mainly conducted among American populations (38 studies; 74.5%); other studies included European (10 studies; 20%) and Asian (3 studies; 5.8%) populations. Most studies used alkylating agents as monotherapy or as part of combination therapy.^17^, ^18^, ^20^, ^21^, ^38^, ^44^, ^45^, ^46^, ^57^, ^61^ The antimetabolite alone or with taxane,^30^, ^37^, ^38^, ^47^, ^48^, ^53^, ^59^ anthracycline alone or with alkylating agents,^15^, ^18^, ^24^, ^30^, ^31^, ^35^, ^51^, ^52^, ^54^ and PKIs were used as monotherapy.^41^, ^42^, ^43^, ^56^, ^58^, ^59^, ^60^ Alkylating agents were commonly used in combination and monotherapy regimens in 13 and 11 studies, respectively. The alkylating agents were mostly combined with anthracycline in 11 studies. The second most common combination evaluated was gemcitabine with docetaxel (8 studies). Common monotherapies were alkylating agents (11 studies) and PKIs (8 studies) (Table 1).

**TABLE 1:** Main characteristics of the studies included in this meta‐analysis and meta‐regression analysis.

Author (Year) Ref.	Country /Continent	Age (median)	Regimen	Sample size (n)	Response rate (n)						mPFS (m)	OS (m)	NOS
					CR	PR	SD	PD	ORR (%)	DCR (%)			
Azizi (1979)^13^	US/NA	52.3	VAD	6	3	1	2	0	66.6	100	NR	NR	6
Hannigan(1983)^14^	US/NA	54	VDaCy	14	3	1	NR	NR	28.5	NR	NR	19.3	7
Omura (1983)^(^ ^15^ ^)a^	US/NA	60	A	28	2	5	5	16	25	43	5.5	7.7	8
Omura (1983)^15^ ^b^	US/NA	61	AD	20	1	5	5	9	30	55	6	7.3	8
Thigpen (1985)^16^	US/NA	54	Pz	11	0	1	1	9	9	18	NR	2	7
Thigpen (1986)^17^	US/NA	54.8	P	19	1	0	7	11	5.3	42	NR	NR	6
Hawkins (1990)^18^ ^a^	EN/EU	47	AI	10	1	0	3	6	10	402	NR	NR	7
Hawkins (1990)^18^ ^b^	EN/EU	53	I	8	0	1	2	5	12.5	37.5	NR	NR	7
Muss (1990)^19^	US/NA	54	MX	12	0	0	2	10	0	16.6	1.4	4.1	7
Thigpen (1991)^20^	US/NA	53	P	33	0	1	18	14	3	57.5	NR	NR	6
Sutton (1992)^21^	US/NA	53	I	35	0	6	10	19	17	45.7	NR	6	7
Currie (1996)^22^	US/NA	55.6	HUDE	38	2	5	20	11	18.5	71	NR	15	7
Thigpen (1996)^23^	US/NA	54	E	28	0	0	13	15	0	46.4	2.1	9.2	7
Sutton (1996)^24^	US/NA	55	AI	33	1	9	17	6	30	81	NR	9.6	7
Resnik (1996)^25^	US/NA	49	AEP	7	1	1	5	0	28.6	100	25.5	43.1	7
Rose (1998)^26^	US/NA	54	E	29	0	2	NR	NR	6.9	6.9	2.1	7.6	7
Sutton (1999)^27^	US/NA	55	Pt	33	3	0	8	22	9	33.3	11	NR	7
Miller (2000)^28^	US/NA	53	TOPO	36	1	3	12	20	11.1	44.4	NR	NR	6
Smith (2002)^29^	US/NA	53	TX	23	0	1	11	11	4.4	52	2.2	7.2	7
Pearl (2002)^30^	US/NA	55	AID	5	1	1	1	2	40	60	13	43	7
Edmonson (2002)^31^	US/NA	52	AMP	35	3	5	14	13	22.8	62.8	4	6.3	7
Hensley (2002)^32^	US/NA	54	GDt	30	3	13	6	8	55.7	75.8	5.6	17.9	7
Gallup (2003)^33^	US/NA	53.5	Pt	48	2	2	11	33	8.3	31.2	1.5	12.1	7
Look (2004)^76^	US/NA	52.5	G	42	1	8	7	26	21.4	38	NR	NR	7
Sutton (2005)^34^	US/NA	52	A	30	1	4	10	15	16.6	50	NR	NR	6
Long (2005)^35^	US/NA	54.5	AMDP	16	1	4	10	1	31	93.7	5.9	14.2	7
D'Adamo (2005)^36^	US/NA	54	AB	7	0	2	4	1	28.6	85.7	8	16	7
Boyar (2005)^37^	US/NA	60	TMZTha	11	0	1	3	7	11	36	2	9.5	7
Anderson (2005)^38^	US/NA	52	TMZ	12	1	1	3	7	2/12	41.6	NR	NR	6
Hensley (2008)^39^	US/NA	50	GDt	45	3	10	24	8	28.8	82	6.7	14.7	7
Hensley (2008)^40^	US/NA	56.3	GDt	39	2	13	11	13	38.4	66.6	4.4	16.1	7
Hensley (2009)^41^	US/NA	56	SU	23	0	2	7	14	8.7	39	1.54	15.1	7
Maki (2009)^42^	US/NA	55	SF	37	0	1	18	18	2.7	51	3.2	22.4	7
Sleijfer (2009)^43^	DU/EU	56.6	Pb	34	0	1	NR	NR	2.9	NR	3	11.8	8
Judson (2010)^44^	US/NA	53	Td	62	0	11	20	31	17.7	50	2.5	12.1	7
Sanfilippo (2011)^45^	IT/EU	56	Td	66	0	11	23	32	16.6	51.5	3.3	14.4	7
Monk (2012)^46^	US/NA	60	Td	20	0	2	10	8	10	60	5.8	26.1	7
Mackay (2012)^47^	US/NA	58	Aflibercept	37	0	0	15	25	0	40.5	1.8	18.1	7
Yoo (2012)^48^	US/NA	54.8	PtC	8	0	1	1	6	12.5	25	2.23	12.4	6
Pautier (2012)^(^ ^49^ ^)a^	FR/EU	62	GDt	21	0	5	10	6	23.8	71.4	4.7	23	8
Pautier (2012)^49^ ^b^	FR/EU	64	G	21	1	3	9	8	19	61.9	5.5	20	8
Takano (2014)^50^	JA/AS	60	GDt	10	1	2	4	3	30	70	5.4	14	7
Hadoux (2014)^51^	FR/EU	51	API	33	4	12	8	9	48.5	72.7	9.8	27	7
Yamagami (2014)^52^	JA/AS	50	API	6	3	0	2	1	50	83	10.2	NR	7
Duska (2014)^53^	US/NA	56.5	Ixabepilone	21	0	0	4	17	0	19	1.4	7	7
Pautier (2015)^54^	FR/EU	58	ATd	47	0	28	13	6	59.5	87.2	8.3	27.5	7
Seddon (2015)^55^	EN/EU	53	GDt	24	0	8	7	9	33.3	62.5	7.1	17.9	7
Benson (2016)^56^	US/NA	55	Pb	44	0	5	25	14	11.3	68	3	17.5	8
Hensley (2017)^57^ ^a^	US/NA	55	D	88	1	6	26	55	8	37.5	1.5	12.9	8
Hensley (2017)^57^ ^b^	US/NA	54	Td	143	1	16	44	82	11.8	42.6	4	13.4	8
Gelderblom (2017)^58^	DU/EU	56	Pb	24	0	1	5	18	2.5	15	3	11.1	7
Hyman (2017)^59^	US/NA	61	Ab	21	0	0	8	13	0	38	1.7	14.5	7
Kim (2018)^60^	KR/AS	57	Pb	27	1	8	9	9	33	66.6	5.8	20	7
Gadducci (2018)^61^ ^a^	IT/EU	54	GDt	38	5	6	15	12	29	68.5	6.9	36.7	8
Gadducci (2018)^61^ ^b^	IT/EU	60	Td	115	8	19	43	45	23.5	61	4.1	20.6	8
Sunar (2019)^62^	TU/AS	53	Pb	28	0	4	17	7	14.3	75	5.2	11.4	8

*Note*: ^a^ and ^b^ denote the two armed studies.

Abbreviations: A, Adriamycin; AB, Adriamycin + Bevacizumab; Ab, Alisertib; ABl, Adriamycin + Bleomycin; AC, Adriamycin + Carboplatin; AD, Adriamycin + Dacarbazine; AEP, Adriamycin + Etoposide + cisplatin; AI, Adriamycin + Ifosfamide; AIDP, Adriamycin + Ifosfamide + Dacarbazine + Cisplatin; AIP, Adriamycin + Cisplatin + Ifosfamide; AMD, Adriamycin + Mitomycin + Dacarbazine: AMP, Adriamycin + Mitomycin + Cisplatin; AS, Asian; AT, Adriamycin + Trabectedin; B, Bevacizumab; Bl, Bleomycin; C, Carboplatin; CR, Complete response; Cy, Cyclophosphamide; D, Dacarbazine; Da, actinomycin‐D; DCR, Disease control rate; Dt, Docetaxel; DU, The Netherlands (Dutch); E, Etoposide; EN, England; EU, Europe; FR, France; G, Gemcitabine; GDt, Gemcitabine + Docetaxel; GDtB, Gemcitabine + Docetaxel + Bevacizumab; GE, Germany; HU, Hydroxyurea; HUDE, Hydroxyurea + Dacarbazine + Etoposide; I, Ifosfamide; IT, Italy; JA, Japan; KR, Korea; M, Mitomycin; m, months; N, Mitoxantrone; NA, North America; NOS, New Casttle Ottawa Scale; NR, Not reported; ORR, Overall response rate; OS, Overall survival; Pb, Pazopanib; PD, Progressive disease; PFS, Progression free survival; PR, Partial response; Pt, Paclitaxel; PtC, Paclitaxel + Carboplatin; Pz, Piperazinedione; SD, Stable disease; SF, Sorafenib; SU, Sunitinib; T, Trabectedin; Tha, Thalidomide; TMZ, Temozolomide; TOPO, Topotecan; TU, Turkey; TX, Trimetrexate; US, United States; V, Vincristine; VAD, Vincristine + Adriamycin + Dacarbazine; VDaCy, Vincristine + actinomycin‐D + Cyclophosphamide.

### 3.2 Quality assessment

The quality of the 51 reports was evaluated by NOS protocols and the QUADAS‐2 quality evaluation standards of the Cochrane reviewer handbook. The detailed quality assessment according to the NOS score is summarized in Table S4. All studies were of high quality, with a score of 7 or more out of 10 (mean score, 7 points). All parameters of the QUADAS‐2 assessment of the bias risk (Figure S1A) and applicability concerns (Figure S1B) are presented. More than half (58%) of the included studies had a low risk of bias for most parameters and applicability concerns. As shown in Figure S1, no significant bias and applicability concerns were found for any selected studies.

### 3.3 Meta‐analysis outcomes

#### 3.3.1 Overall efficiency of systemic therapy

**FIGURE 2:**
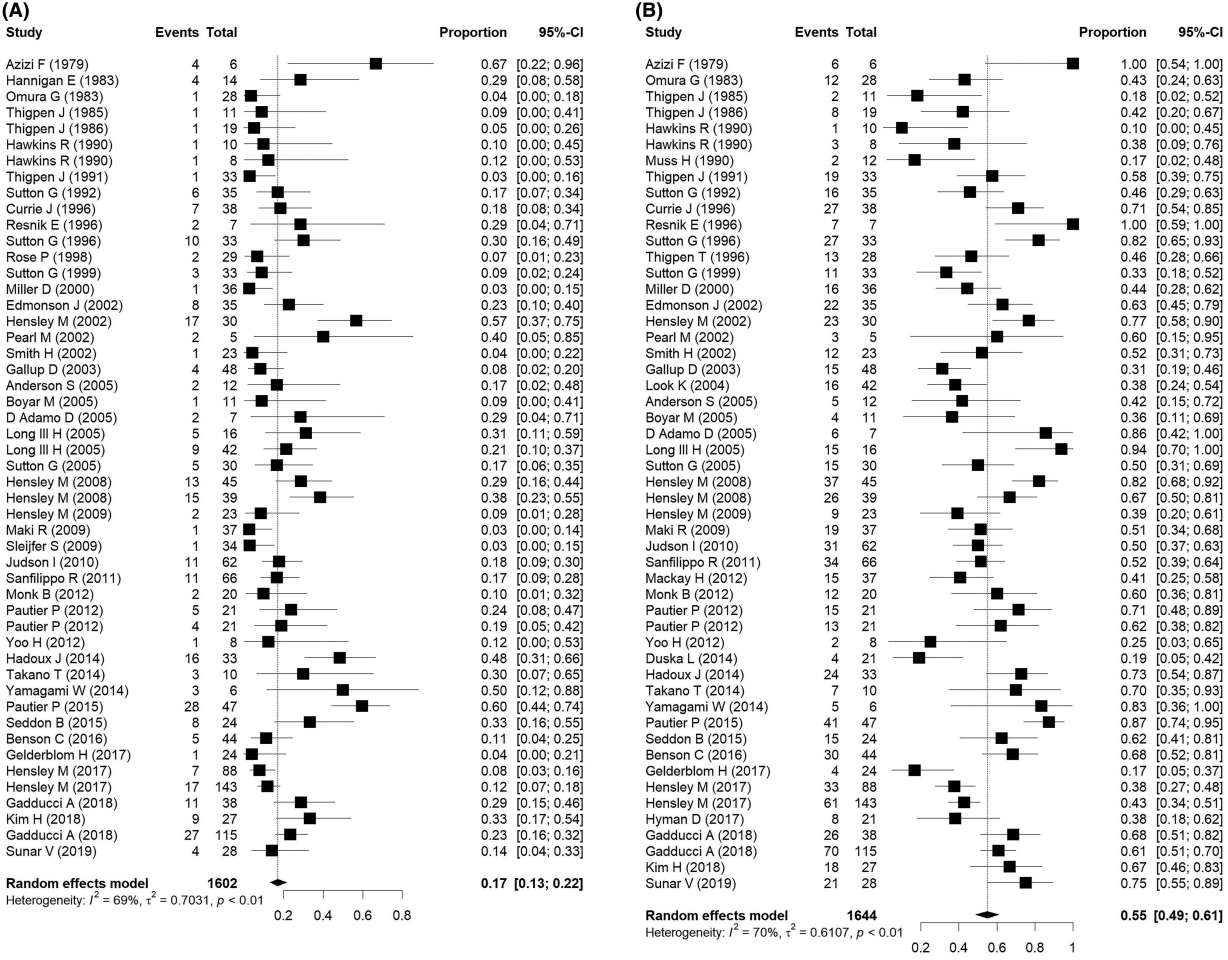
Forest plots of the objective response rates (ORRs) and disease control rates (DCRs). (A). Overall pooled ORR of the 45 studies indicated high heterogeneity for the ORR of advanced uterine leiomyosarcoma patients. (B) Overall pooled DCR of 48 studies of advanced uterine leiomyosarcoma (uLMS). Heterogeneity is the variability between the study‐specific effects that a random variation cannot explain. CI, confidence interval.

We used the pooled proportions test method to compare the efficiency of different treatment regimens (based on the ORR and DCR parameters) for advanced uLMS. Because study heterogeneity identified by the fixed effects model was high overall, we used the random effects approach. The pooled ORR was 0.17 (95% confidence interval [CI]: 0.13–0.22) and the pooled DCR was 0.55 (95% CI: 0.49–0.61) (*p* < 0.01). Based on the heterogeneous cross of studies of the ORR and DCR of different treatment regimens, both parameters appeared to be associated with greatly improved prognoses with some regimens for patients with metastatic uLMS (Figure 2A,B). An overall heterogeneous estimation performed separately for the monotherapy and combination chemotherapy regimens also showed significant differences in the prognoses of patients with advanced uLMS (Figure S2A,B).

#### 3.3.2 Subgroup analyses

A subgroup analysis was conducted to evaluate the influence of prognostic factors (monotherapy or combination therapy, first‐line or second‐line therapy, study design, and disease stage) on the ORR and DCR (Table 2). Initially, these factors were tested using univariate models. Then, predictors were added to multivariate models to investigate their contribution to any residual heterogeneity. The results of the random effects model revealed variables that contribute to overall heterogeneity (*p* < 0.05).

**TABLE 2:** Results of the meta‐analysis of the association between the subgroups and outcomes of patients with uterine leiomyosarcoma.

		Proportion	No. of studies	Pooled	OR (95% CI)	*Q*‐value	*I* ^2^ (%)	*p*‐value
ORR	Stage	I–III	6	0.21	(0.13–0. 33)	5	0.00	0.42
		III–IVb	44	0.17	(0.13–0. 22)	153.84	72.00	<0.01
	Study design	Retrospective	11	0.21	(0.14–0. 31)	26.22	62.00	<0.01
		Phase II	39	0.16	(0.12–0. 21)	132.47	71.30	<0.01
	Mono therapy	Alkylating agents	11	0.14	(0.11–0.17)	15.82	36.80	0.65
		Anthracycline	3	0.17	(0.10–0.27)	0.61	0.00	0.28
		Topoisomerase inhibitors	4	0.06	(0.03–0.13)	0.34	0.00	0.22
		Antimetabolite	3	0.16	(0.09–0.27)	2.71	26.30	0.82
		Taxane	2	0.08	(0.04–0.17)	0.01	0.00	<0.01
		Protein kinase inhibitors	8	0.06	(0.06–0.13)	12.87	45.60	0.68
	Dual therapy	Anthracycline + Alkylating agents	8	0.36	(0.32–0.46)	17.02	58.90	0.06
		Antimetabolite + Taxane	8	0.33	(0.27–0.40)	10.13	30.90	<0.01
	Triple therapy	Anthracycline + Alkylating agent + Vinca alkaloid	2	0.20	(0.77–0.42)	0	0.00	0.56
	Firstst line	Monotherapy	7	0.09	(0.06–0.14)	3.92	0.00	0.47
		Combination therapy	12	0.35	(0.30–0.41)	23.01	52.20	<0.01
	Second line	Monotherapy	24	0.12	(0.10–0.14)	31.12	26.10	0.02
		Combination therapy	7	0.25	(0.18–0.33)	3.54	0.00	<0.01
	Tumor status	Recurrent	21	0.15	(0.10–0.21)	46.53	57.00	<0.01
		Metastatic	23	0.22	(0.16–0.35)	102.44	78.50	<0.01
		Persistent	8	0.14	(8.14–24.43)	14.71	52.40	<0.01
	Organ involved	Bone	7	0.21	(0.12–0.36)	34.85	83.00	<0.01
		Peritoneal	13	0.23	(0.17–0.30)	21.95	45.30	0.04
		NR	15	0.17	(0.11–0.25)	51.09	73.00	<0.01
		Respiratory	12	0.16	(0.08–0.29)	34.09	67.70	<0.01
		Extra‐pelvic	5	0.07	(0.02–0.18)	9	56.00	0.06
	Disease evaluation	CT	19	0.24	(0.18–0.31)	38.36	53.10	<0.01
		CXR	7	0.21	(0.12–0.34)	11.83	49.30	0.07
		MRI	7	0.26	(0.15–0.42)	35.15	83.00	<0.01
		NR	6	0.12	(0.09–0.16)	8.47	41.00	0.13
		Radiographic	13	0.09	(0.05–0.15)	31.6	62.00	<0.01
DCR	Stage	I–III	6	0.66	(0.41–0.85)	8.73	43.00	0.12
		III–IVb	46	0.55	(0.48–0.61)	161.97	72.20	<0.01
	Study design	Retrospective	10	0.58	(0.46–0.70)	26.46	66.00	<0.01
		Phase II	42	0.54	(0.47–0.61)	142.51	71.20	<0.01
	Mono therapy	Alkylating agents alone	11	0.48	(0.44–0.52)	16.47	39.30	<0.01
		Anthracycline alone	3	0.41	(0.30–0.53)	3.53	43.40	<0.01
		Topoisomerase inhibitors	3	0.41	(0.30–0.52)	2.59	22.90	<0.01
		Antimetabolite alone	3	0.47	(0.37–0.58)	3.37	40.60	<0.01
		Taxane alone	2	0.32	(0.22–0.42)	0.04	0.00	<0.01
		Protein kinase inhibitors	8	0.45	(0.39–0.52)	24.37	71.30	<0.01
	Dual therapy	Antimetabolite + Taxane	8	0.70	(0.63–0.75)	10.3	32.00	<0.01
		Anthracycline + Alkylating	8	0.74	(0.67–0.79)	10.21	31.40	<0.01
	Triple therapy	Anthracycline + Alkylating agent + Vinca alkaloid	2	0.30	(0.14–0.52)	0	0.00	0.89
	First line	Monotherapy	7	0.47	(0.40–0.54)	0	0	<0.01
		Combination therapy	12	0.72	(0.66–0.76)	12.81	14.10	<0.01
	second line	Monotherapy	7	0.66	(0.58–0.73)	9.15	34.40	<0.01
		Combination therapy	23	0.39	(0.31–0.47)	0.5302	0.7281	0.02
	Tumor status	Recurrent	19	0.55	(0.47–0.63)	47.75	62.30	<0.01
		Metastatic	23	0.57	(0.47–0.67)	96.67	77.20	<0.01
		Persistent	12	0.52	(0.42–0.62)	33.39	67.10	<0.01
	Organ involved	NR	15	0.56	(0.42–0.69)	52.47	73.30	<0.01
		Bone	7	0.60	(0.50–0.68)	10.12	41.00	0.12
		Respiratory	13	55.21	(0.42–0.67)	36.62	67.20	<0.01
		Peritoneal	12	0.62	(0.51–0.72)	48.26	77.20	<0.01
		Extra‐pelvic	7	0.45	(0.34–0.56)	11.89	50.00	0.06
	Disease evaluation	CT	18	0.61	(0.51–0.70)	65.31	74.00	<0.01
		CXR	8	0.49	(0.26–0.72	17.35	60.00	0.02
		MRI	8	0.55	(0.45–0.65)	17.04	60.00	0.02
		NR	6	0.54	(0.39–0.68)	12.22	59.10	0.03
		Radiographic	14	0.52	(0.41–0.63)	45.79	71.60	<0.01

Abbreviations: CI, confidence interval; CT, computed tomography; CXR, chest X‐ray; MRI, magnetic resonance imaging; NR, not reported; USG, ultrasonography.

The pooled ORRs for the phase II and retrospective study designs were 16% (95% CI: 0.12–0.21) and 21% (95% CI: 0.14–0.31), respectively (Figure S3A). Among treatment regimens, dual therapy showed a significant pooled ORR of 32% (95% CI: 0.24–0.41). Anthracycline plus alkylating agents showed a pooled ORR of 39.5% (95% CI: 0.32–0.46); however, for patients with FIGO stage IVb, the pooled ORR was 17% (95% CI: 0.13–0.22). These data showed that patients with advanced‐stage disease benefited more from treatment when used as first‐line therapy (pooled ORR = 35.6%; 95% CI: 0.30–0.41; *p* < 0.01) (Figure S3DB–). Moreover, the pooled ORR for monotherapy favored PKIs when used as second‐line therapy (Table 2).

Additionally, the subgroup analysis of the DCR showed significant heterogeneity of the study designs and disease stages (*p* < 0.01) (Figure S4A). Dual therapy comprising anthracycline plus alkylating agents (pooled DCR = 74%; 95% CI: 0.67–0.80) and that comprising antimetabolite plus taxane (pooled DCR = 70%; 95% CI: 0.63–0.75) resulted in higher therapeutic prognostic values (Figure S4B). Table 2 shows that monotherapy with alkylating agents (pooled DCR = 48%; 95% CI: 0.44–0.52), antimetabolites (pooled DCR = 47%; 95% CI: 0.37–0.58), and PKIs (pooled DCR = 45%; 95% CI: 0.39–0.52) significantly improved the prognosis (*p* < 0.01) (Figure S4C). Chemotherapy regimens as monotherapy and in combination were more efficacious when used as first‐line therapy (pooled DCR = 47%; 95% CI: 0.40–0.54) rather than second‐line therapy (pooled DCR = 72%; 95% CI: 0.66–0.76). However, combination therapy was almost equally beneficial as chemotherapy when used as second‐line therapy (pooled DCR = 66%; 95% CI: 0.58–0.73). Therefore, the monotherapies were not as favorable as second‐line therapies in terms of improving the prognosis of patients with late‐stage uLMS (Table 2) (Figure S4D,E).

By analyzing the DCR as an endpoint, we found that magnetic resonance imaging (MRI) and computed tomography (CT) are the best monitoring methods because of their high diagnostic accuracy for uLMS disease surveillance. Among disease evaluation tools, CT provided the best diagnostic accuracy (pooled DCR = 61%; 95% CI: 0.51–0.70). We observed significantly increased heterogeneity for patients with late FIGO stages (pooled DCR = 55%; 95% CI: 0.48–0.61; *p* < 0.01) (Figure S4F). Interestingly, patients with peritoneal metastasis had better therapeutic prognoses than those without (pooled DCR = 62%; 95% CI: 0.51–0.72; *p* < 0.01). Overall, our results showed that ORR and DCR were significantly correlated with higher FIGO stages, phase II study designs, dual‐regimen therapy types, bone and peritoneum involvement, surveillance tools including MRI and CT, and metastatic tumors (Table 2).

### 3.4 Meta‐regression analysis outcomes

#### 3.4.1 Effects of clinicopathologic factors on the overall response rate

**FIGURE 3:**
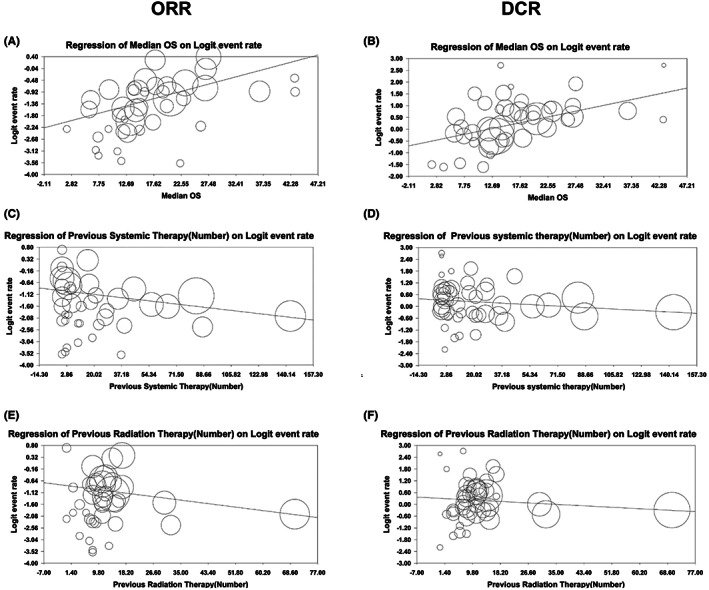
Meta‐regression plot used to evaluate the effect on the objective response rate (ORR) and disease control rate (DCR) of advanced uterine leiomyosarcoma (uLMS) patients (A,B). Median overall survival with (C,D) neoadjuvant chemotherapy (NACT) and (E,F) previous radiation therapy. The size of each square is proportional to the ratio of the weighted percentage of each study to the standardized mean difference (SDM). Weights were retrieved from the random effects analysis.

The meta‐regression analysis showed an association between OS, previous NACT, or local radiotherapy among uLMS patients with earlier FIGO stages (Figure 3A,C,E). These results suggest that OS, a history of NACT, and local radiotherapy significantly influence the ORR of therapies for patients with advanced uLMS (*p* < 0.001) (Table 3). According to the results of the meta‐regression analysis, more of the included patients in the present trial had a history of radiation and systemic therapy for their early‐stage disease. The current treatment for advanced‐stage disease positively impacted the ORR (*p* < 0.05). However, an increased ORR was associated with improved OS. Moreover, we did not find a significant correlation between the ORR and the article publication year and median patient age (*p* > 0.05) (Figure S5A,B).

**TABLE 3:** Results of the meta‐regression analysis of the included studies.

	Parameters	Regression coefficient	Lower limit	Upper limit	*p*‐value
ORR	Median age	0.00	−0.04	0.04	0.90
	Median OS	0.05	0.04	0.07	<0.001
	Year of publication	0.01	−0.00	0.02	0.14
	Previous systemic therapy	−0.00	−0.01	−0.00	<0.001
	Previous radiation therapy	−0.01	−0.02	−0.00	<0.001
DCR	Median age	0.01	−0.02	0.04	0.53
	Median OS	0.04	0.03	0.06	<0.001
	Year of publication	0.01	−0.00	0.02	0.052
	Previous systemic therapy	−0.00	−0.00	−0.002	<0.001
	Previous radiation therapy	−0.00	−0.01	−0.00	0.003

Abbreviations: DCR, disease control rate; ORR, overall response rate; OS, overall survival;

#### 3.4.2 Effects of clinicopathologic factors on the disease control rate

The meta‐regression analysis of the DCR demonstrated the favorable prognostic values of OS, NACT, and local radiotherapy in terms of the outcomes of patients with advanced uLMS (Figure 3 and Table 3). This finding indicated the significant impact of OS (Figure 3B), history of NACT (Figure 3D), and previous radiotherapy (Figure 3F) on the DCR of patients with uLMS (*p* < 0.01). However, an increase in the OS was associated with a better DCR. Additionally, the publication year and median patient age did not improve the prognostic value of the DCR (*p* > 0.05) (Figures S5C,D).

### 3.5 Publication bias

Diffusion bias was evaluated to determine the consequences of the DCR and ORR omission bias and to estimate the number of missing studies (Figure S6). Based on the regression results, diffusion was not significant to the DCR outcome (*t* = 0.77; *p =* 0.44) (Figure S6A), and the omission bias of the ORR outcome was statistically significant (*t* = 3.31; *p* = 0.001) (Figure S6B). To modify the effect of missed studies using the trim‐and‐fill method, adjusted ORR estimates were used (Figure S6C). Consequently, 13 studies were estimated as missing. Additionally, the adjusted ORR value was 26.45 (95% CI: 24.16–28.87).

## 4 DISCUSSION

We present the first comprehensive meta‐analysis and meta‐regression analysis to compare the impact of systemic therapeutic approaches on advanced, metastatic, and relapsing uLMS. Our results showed a significantly improved ORR across studies for dual‐regimen chemotherapy (anthracycline plus alkylating therapy) when used as first‐line therapy. Our findings suggested that dual‐therapy regimens (anthracycline plus alkylating or antimetabolite therapy plus taxane) and PKIs for patients with late‐stage FIGO III–IVb disease significantly increased the DCR. Furthermore, among the disease‐measuring tools, CT, and MRI are beneficial.

NACT after surgery is emerging as an alternative treatment strategy for advanced uLMS. A recent systematic review showed a significant logistic regression correlation coefficient between a history of NACT and an improved prognosis for advanced uLMS.^63^ The finding of a phase II clinical trial suggested the use of oral gefitinib as first‐line treatment for recurrent uLMS with pooled progression‐free survival (PFS). Randomized controlled trials reported the benefits of localized chemoradiotherapy as second‐line therapy for patients with advanced uLMS. NACT combined with radiotherapy can predict optimal secondary cytoreduction. Furthermore, a reduced overall risk of uLMS relapse with distant metastasis was observed with NACT and previous radiotherapy (*p* < 0.001). This finding suggests that patients who receive chemoradiotherapy for local spread during earlier stages of disease experience significant improvement with later treatment when disease relapses with metastasis to distant organs.

Alkylating agents, such as doxorubicin and rituximab, have potential efficacy for uLMS when used as monotherapy or combination therapy.^64^, ^65^ Doxorubicin is a commonly used anthracycline drug that has an acceptable ORR or PFS when used as combination therapy for uLMS.^6^, ^66^ Recently, a large retrospective study showed that the combination of doxorubicin and dacarbazine as first‐line chemotherapy for advanced uLMS was more effective than anthracycline alone.^65^, ^67^ Our study also demonstrated that anthracycline plus alkylating chemotherapy as first‐line treatment can be used effectively for patients with late FIGO stages of uLMS.^66^ Trabectedin, which is an alkylating agent, has also shown some efficacy when used with Adriamycin as first‐line chemotherapy for advanced uLMS.^54^, ^68^ Nevertheless, prospective studies are required to assess the efficacy of adding alkylating agents to anthracycline‐based chemotherapy.

Gemcitabine is a common antimetabolite agent that is used as second‐line therapy for advanced solid tumors; it is more commonly used as combination therapy with docetaxel than alone.^39^ Phase III, randomized, controlled trials showed the efficacy of gemcitabine‐docetaxel as first‐line treatment for advanced uLMS.^40^ Among the aromatase inhibitors, letrozole was the only treatment that showed significant ORR values during a single‐arm, phase II, clinical trial.^69^ However, second‐line chemotherapy with other aromatase inhibitors, such as alisertib,^59^ nivolumab,^70^ and sunitinib,^41^ did not have significant effects on the PFS and DCR of advanced uLMS.

PKIs, as second‐line monotherapies, are also effective for the treatment of advanced uLMS. Recent studies showed that second‐line therapy with temozolomide plus thalidomide and that with Adriamycin plus bleomycin do not have statistical efficacy for the treatment of early‐stage uLMS. However, other studies observed the significant prognostic value of Adriamycin plus bleomycin for the treatment of metastatic uLMS.^37^, ^38^ Systemic therapy with anthracycline plus alkylating agents combined with either vinca alkaloid or the topoisomerase inhibitor had some prognostic benefits, but further trials are required to statistically establish the benefit of this combination therapy for patients with uLMS.^13^, ^14^, ^22^, ^24^ Moreover, it is well‐accepted that aflibercept and ixabepilone monotherapies are effective second‐line therapies for advanced uLMS.^47^, ^53^ More recently, an investigation of the *BRCA1/2* gene mutation in uLMS revealed *BRCA2* mutations; therefore, PARP inhibitors are undergoing testing and have shown some efficacy.^70^, ^71^, ^72^ However, further clinical studies are necessary until the desired responses are achieved.

The pooled PFS was considered a standard endpoint index for measuring the efficiency of combination therapy during randomized controlled trials. Currently, the lack of standard response evaluation criteria for early‐phase trials is the main reason for this discrepancy. However, we made an effort to evaluate the outcomes of patients who received various systemic treatment regimens based on RECIST. We studied reports of 1664 patients with uLMS that included the ORR and DCR as eligible primary endpoints. Our data showed that the ORR and DCR as endpoints were more reliable because of their availability in the included studies. Therefore, during a single‐arm, phase II trial that evaluated the effectiveness of molecular‐targeted therapy, these can be considered a reliable predictor, but it is possible that the long‐lasting lesion maintenance effects of drugs cannot be detected by non‐metric endpoint indexes.^73^ Additionally, during a single‐arm, phase II trial, it is crucial to set the primary endpoint to assess a significant response.

The meta‐analysis and meta‐regression analysis are complementary methods that are often used together in research studies to evaluate the efficacy and safety of different treatment regimens. A meta‐analysis provides a quantitative synthesis of the results of multiple studies, which can help to identify the overall effect size of the intervention and heterogeneity among studies. In contrast, a meta‐regression analysis allows the assessment of the influence of study‐specific variables on the treatment effect, such as patient demographics or study design. By combining these two methods, it is possible to obtain a more comprehensive understanding of the factors that influence the treatment response of patients with uLMS and provide more reliable evidence‐based recommendations for clinical practice. Moreover, the use of a meta‐regression analysis can help to identify potential sources of bias and confounding factors that may affect the study results, which can be used to improve the design and reporting of future studies in this field. Therefore, the use of both a meta‐analysis and meta‐regression analysis is essential to draw more accurate conclusions from the available evidence and guiding the development of effective treatment strategies for patients with uLMS. Our findings showed that CT and MRI are beneficial monitoring options for evaluating treatment. Retrospective studies have confirmed the prognostic relevance of positron emission tomography/CT imaging for staging, restaging, monitoring the response to therapy, and surveillance of patients with uLMS.^74^ However, because of the small number of studies that have used positron emission tomography/CT, we could not confirm this premise. Conversely, our analysis showed that CT has high diagnostic accuracy and is the most accurate method for monitoring the response to therapy by patients with advanced uLMS.

To our best knowledge, this is the first systematic study to address advanced or metastatic unresectable uLMS using a large patient database. We believe that our observations provide a basis for designing efficient therapeutic strategies and direct clinical practices to improve the outcomes of patients with advanced, metastatic, and relapsing uLMS.^75^ In addition to the urgent clinical need for the development of new drug therapies, especially for patients with a history of chemotherapy (two or more lines), there is also a need for clinical trials based on pharmacogenomics and biomarker‐based patient selection.

Some limitations of the present investigation should be addressed. First, we only searched for works written in English. Second, many studies did not provide sufficient detailed information to calculate the adjusted estimates; therefore, our results were based on unadjusted estimates. The efficiency of postoperative therapy for uLMS remains controversial.^1^ We propose that future research should explore further factors that significantly impact the outcomes of disease and study treatment responses on a larger scale. Undoubtedly, international platforms should establish clinical trials, thus replacing single‐institution studies, to set an open standard. Future research should also further unravel which factors contribute to heterogeneity across studies. Third, the included clinical trials had a single‐arm nature, which hampers the distinction between the effect of the treatment and the impact of the medical history. Therefore, it is possible that the study did not include all the potential confounding factors even though we performed subgroup analyses to control for several possible confounders. The ORR and DCR should be interpreted with a frame of reference for comparison in this specific setting. The main goal of a clinical trial is to provide evidence of the improved quality of life through a better response to a particular therapeutic approach.

## 5 CONCLUSIONS

In conclusion, first‐line monotherapy with anthracycline and dual therapy with anthracycline plus alkylating agents and second‐line monotherapy with PKIs and antimetabolites and a dual regimen of antimetabolites plus taxane appear to be effective treatments for advanced‐stage uLMS with distant metastases. Additionally, NACT and localized radiotherapy are positively associated with an improved prognosis for relapsed uLMS. Large, prospective, randomized, multi‐institutional trials are required to achieve a better understanding of the value of various treatments.

## AUTHOR CONTRIBUTIONS

**Iqra Ijaz:** Conceptualization (lead); data curation (lead); formal analysis (lead); funding acquisition (equal); methodology (lead); project administration (supporting); writing – review and editing (lead). **Muhammad Naveed Shahzad:** Conceptualization (equal); data curation (equal); methodology (equal); writing – review and editing (equal). **Hossein Hosseinifard:** Formal analysis (equal); software (equal); validation (equal). **Shuya Liu:** Formal analysis (equal); resources (equal); validation (equal); writing – review and editing (equal). **Masoud Ostadi Sefidan:** Formal analysis (equal); software (equal); validation (equal); writing – review and editing (equal). **Lubna Ejaz Kahloon:** Conceptualization (equal); data curation (equal); methodology (equal); writing – review and editing (equal). **Saber Imani:** Formal analysis (supporting); software (equal); validation (equal); visualization (equal); writing – review and editing (equal). **Zhong Hua:** Funding acquisition (equal); project administration (equal); supervision (equal); visualization (equal); writing – original draft (equal); writing – review and editing (equal). **Qin Zhang Yu:** Project administration (equal); resources (equal); supervision (equal); visualization (equal); writing – original draft (equal); writing – review and editing (equal).

## CONFLICT OF INTEREST STATEMENT

All authors declare no conflicts of interest.

## FUNDING STATEMENT

This work was supported by a grant from the university‐level online education and teaching reform of Southwest Medical University, Luzhou City, China (grant number 2020XSJG‐C02‐18) and a grant from the special research project of the Health Commission of Luzhou City (HCLC), Sichuan Province, China.

## ETHICS STATEMENTS

This meta‐analysis was approved by an independent ethics committee/institutional review board of the Sichuan Provincial Center for Gynecological and Breast Diseases at Southwest Medical University.

## Supporting information


**Data S1:** Supporting information

## Data Availability

The original contributions presented in the study are included in the article/supplementary material; further inquiries can be directed to the corresponding authors.
