# Supplementary material for: Evaluation of the efficacy of systemic therapy for advanced uterine leiomyosarcoma: A systematic review, meta‐analysis, and meta‐regression analysis
Source: Cancer Med. 2023 Apr 20;12(13):13894–911. doi: 10.1002/cam4.5930 (PMC10358251; doi:10.1002/cam4.5930)

Supplementary Material

# Supplementary Table S 1: Included and excluded studies in meta-analysis and meta regression analysis of Overall Respose Rate (ORR)

# Included studies

| 1. | Azizi F, Bitran J, Javehari G, Herbst AL. Remission of uterine leiomyosarcomas treated with vincristine, Adriamycin, and dimethyl-triazeno-imidazole carboximide. *Am J Obstet Gynecol*. 1979;133(4):379-381. doi:10.1016/0002-9378(79)90055-3 |
| --- | --- |
| 2. | Hannigan E V., Freedman RS, Elder KW, Rutledge FN. Treatment of advanced uterine sarcoma with vincristine, actinomycin D, and cyclophosphamide. *Gynecol Oncol*. 1983;15(2):224-229. doi:10.1016/0090-8258(83)90078-1 |
| 3. | Omura GA, Major FJ, Blessing JA, et al. A randomized study of adriamycin with and without dimethyl triazenoimidazole carboxamide in advanced uterine sarcomas. *Cancer*. 1983;52(4):626-632. doi:10.1002/1097-0142(19830815)52:4<626::aid-cncr2820520409>3.0.co;2-e |
| 4. | Thigpen JT, Blessing JA, Homesley HD, Hacker N, Curry SL. Phase II trial of piperazinedione in patients with advanced or recurrent uterine sarcoma. A Gynecologic Oncology Group study. *Am J Clin Oncol*. 1985;8(5):350-352. doi:10.1097/00000421-198510000-00002 |
| 5. | Thigpen JT, Blessing JA, Wilbanks GD. Cisplatin as second-line chemotherapy in the treatment of advanced or recurrent leiomyosarcoma of the uterus. A phase II trial of the Gynecologic Oncology Group. *Am J Clin Oncol*. 1986;9(1):18-20. doi:10.1097/00000421-198602000-00005 |
| 6. | Hawkins RE, Wiltshaw E, Mansi JL. Ifosfamide with and without adriamycin in advanced uterine leiomyosarcoma. *Cancer Chemother Pharmacol*. 1990;26 Suppl:S26-9. doi:10.1007/BF00685412 |
| 7. | Thigpen JT, Blessing JA, Beecham J, Homesley H, Yordan E. Phase II trial of cisplatin as first-line chemotherapy in patients with advanced or recurrent uterine sarcomas: a Gynecologic Oncology Group study. *J Clin Oncol*. 1991;9(11):1962-1966. doi:10.1200/JCO.1991.9.11.1962 |
| 8 | Sutton GP, Blessing JA, Barrett RJ, McGehee R. Phase II trial of ifosfamide and mesna in leiomyosarcoma of the uterus: a Gynecologic Oncology Group study. *Am J Obstet Gynecol*. 1992;166(2):556-559. doi:10.1016/0002-9378(92)91671-v |
| 9 | Currie J, Blessing JA, Muss HB, Fowler J, Berman M, Burke TW. Combination chemotherapy with hydroxyurea, dacarbazine (DTIC), and etoposide in the treatment of uterine leiomyosarcoma: a Gynecologic Oncology Group study. *Gynecol Oncol*. 1996;61(1):27-30. doi:10.1006/gyno.1996.0091 |
| 10 | Sutton G, Blessing JA, Malfetano JH. Ifosfamide and doxorubicin in the treatment of advanced leiomyosarcomas of the uterus: a Gynecologic Oncology Group study. *Gynecol Oncol*. 1996;62(2):226-229. doi:10.1006/gyno.1996.0220 |
| 11 | Resnik E, Chambers SK, Carcangiu ML, Kohorn EI, Schwartz PE, Chambers IT. Malignant uterine smooth muscle tumors: role of etoposide, cisplatin, and doxorubicin (EPA) chemotherapy. *J Surg Oncol*. 1996;63(3):145-147. doi:10.1002/(SICI)1096-9098(199611)63:3<145::AID-JSO3>3.0.CO;2-D |
| 12 | Rose PG, Blessing JA, Soper JT, Barter JF. Prolonged oral etoposide in recurrent or advanced leiomyosarcoma of the uterus: a gynecologic oncology group study. *Gynecol Oncol*. 1998;70(2):267-271. doi:10.1006/gyno.1998.5080 |
| 13 | Sutton G, Blessing JA, Ball H. Phase II trial of paclitaxel in leiomyosarcoma of the uterus: a gynecologic oncology group study. *Gynecol Oncol*. 1999;74(3):346-349. doi:10.1006/gyno.1999.5463 |
| 14 | Miller DS, Blessing JA, Kilgore LC, Mannel R, Van Le L. Phase II trial of topotecan in patients with advanced, persistent, or recurrent uterine leiomyosarcomas: a Gynecologic Oncology Group Study. *Am J Clin Oncol*. 2000;23(4):355-357. doi:10.1097/00000421-200008000-00009 |
| 15 | Smith HO, Blessing JA, Vaccarello L. Trimetrexate in the treatment of recurrent or advanced leiomyosarcoma of the uterus: a phase II study of the Gynecologic Oncology Group. *Gynecol Oncol*. 2002;84(1):140-144. doi:10.1006/gyno.2001.6482 |
| 16 | Pearl ML, Inagami M, McCauley DL, Valea FA, Chalas E, Fischer M. Mesna, doxorubicin, ifosfamide, and dacarbazine (MAID) chemotherapy for gynecological sarcomas. *Int J Gynecol Cancer*. 2002;12(6):745-748. doi:10.1046/j.1525-1438.2002.01139.x |
| 17 | Edmonson JH, Blessing JA, Cosin JA, Miller DS, Cohn DE, Rotmensch J. Phase II study of mitomycin, doxorubicin, and cisplatin in the treatment of advanced uterine leiomyosarcoma: a Gynecologic Oncology Group study. *Gynecol Oncol*. 2002;85(3):507-510. doi:10.1006/gyno.2002.6661 |
| 18 | Hensley ML, Maki R, Venkatraman E, et al. Gemcitabine and docetaxel in patients with unresectable leiomyosarcoma: results of a phase II trial. *J Clin Oncol*. 2002;20(12):2824-2831. doi:10.1200/JCO.2002.11.050 |
| 19 | Gallup DG, Blessing JA, Andersen W, Morgan MA, Gynecologic Oncology Group Study. Evaluation of paclitaxel in previously treated leiomyosarcoma of the uterus: a gynecologic oncology group study. *Gynecol Oncol*. 2003;89(1):48-51. doi:10.1016/s0090-8258(02)00136-1 |
| 20 | Sutton G, Blessing J, Hanjani P, Kramer P, Gynecologic Oncology Group. Phase II evaluation of liposomal doxorubicin (Doxil) in recurrent or advanced leiomyosarcoma of the uterus: a Gynecologic Oncology Group study. *Gynecol Oncol*. 2005;96(3):749-752. doi:10.1016/j.ygyno.2004.11.036 |
| 21 | Long HJ, Blessing JA, Sorosky J. Phase II trial of dacarbazine, mitomycin, doxorubicin, and cisplatin with sargramostim in uterine leiomyosarcoma: a Gynecologic Oncology Group study. *Gynecol Oncol*. 2005;99(2):339-342. doi:10.1016/j.ygyno.2005.06.002 |
| 22 | D’Adamo DR, Anderson SE, Albritton K, et al. Phase II study of doxorubicin and bevacizumab for patients with metastatic soft-tissue sarcomas. *J Clin Oncol*. 2005;23(28):7135-7142. doi:10.1200/JCO.2005.16.139 |
| 23 | Boyar MS, Hesdorffer M, Keohan ML, Jin Z, Taub RN. Phase II Study of Temozolomide and Thalidomide in Patients with Unresectable or Metastatic Leiomyosarcoma. *Sarcoma*. 2008;2008:412503. doi:10.1155/2008/412503 |
| 24 | Anderson S, Aghajanian C. Temozolomide in uterine leiomyosarcomas. *Gynecol Oncol*. 2005;98(1):99-103. doi:10.1016/j.ygyno.2005.03.018 |
| 25 | Hensley ML, Blessing JA, Degeest K, Abulafia O, Rose PG, Homesley HD. Fixed-dose rate gemcitabine plus docetaxel as second-line therapy for metastatic uterine leiomyosarcoma: a Gynecologic Oncology Group phase II study. *Gynecol Oncol*. 2008;109(3):323-328. doi:10.1016/j.ygyno.2008.02.024 |
| 26 | Hensley ML, Blessing JA, Mannel R, Rose PG. Fixed-dose rate gemcitabine plus docetaxel as first-line therapy for metastatic uterine leiomyosarcoma: a Gynecologic Oncology Group phase II trial. *Gynecol Oncol*. 2008;109(3):329-334. doi:10.1016/j.ygyno.2008.03.010 |
| 27 | Hensley ML, Sill MW, Scribner DR, et al. Sunitinib malate in the treatment of recurrent or persistent uterine leiomyosarcoma: a Gynecologic Oncology Group phase II study. *Gynecol Oncol*. 2009;115(3):460-465. doi:10.1016/j.ygyno.2009.09.011 |
| 28. | Maki RG, D’Adamo DR, Keohan ML, et al. Phase II study of sorafenib in patients with metastatic or recurrent sarcomas. *J Clin Oncol*. 2009;27(19):3133-3140. doi:10.1200/JCO.2008.20.4495 |
| 29 | Sleijfer S, Ray-Coquard I, Papai Z, et al. Pazopanib, a multikinase angiogenesis inhibitor, in patients with relapsed or refractory advanced soft tissue sarcoma: a phase II study from the European organisation for research and treatment of cancer-soft tissue and bone sarcoma group (EORTC study 620. *J Clin Oncol*. 2009;27(19):3126-3132. doi:10.1200/JCO.2008.21.3223 |
| 30 | Judson IR, Blay J, Chawla SP, et al. Trabectedin (Tr) in the treatment of advanced uterine leiomyosarcomas (U-LMS): Results of a pooled analysis of five single-agent phase II studies using the recommended dose. *J Clin Oncol*. 2010;28(15_suppl):10028-10028. doi:10.1200/jco.2010.28.15_suppl.10028 |
| 31 | Sanfilippo R, Grosso F, Jones RL, et al. Trabectedin in advanced uterine leiomyosarcomas: a retrospective case series analysis from two reference centers. *Gynecol Oncol*. 2011;123(3):553-556. doi:10.1016/j.ygyno.2011.08.016 |
| 32 | Monk BJ, Blessing JA, Street DG, Muller CY, Burke JJ, Hensley ML. A phase II evaluation of trabectedin in the treatment of advanced, persistent, or recurrent uterine leiomyosarcoma: a gynecologic oncology group study. *Gynecol Oncol*. 2012;124(1):48-52. doi:10.1016/j.ygyno.2011.09.019 |
| 33 | Yoo HJ, Lim MC, Lim S, et al. Phase II study of paclitaxel in combination with carboplatin for patients with recurrent or persistent uterine sarcoma. *Arch Gynecol Obstet*. 2012;286(6):1529-1535. doi:10.1007/s00404-012-2466-4 |
| 34 | Pautier P, Floquet A, Penel N, et al. Randomized multicenter and stratified phase II study of gemcitabine alone versus gemcitabine and docetaxel in patients with metastatic or relapsed leiomyosarcomas: a Federation Nationale des Centres de Lutte Contre le Cancer (FNCLCC) French Sarcoma Group. *Oncologist*. 2012;17(9):1213-1220. doi:10.1634/theoncologist.2011-0467 |
| 35 | Takano T, Niikura H, Ito K, et al. Feasibility study of gemcitabine plus docetaxel in advanced or recurrent uterine leiomyosarcoma and undifferentiated endometrial sarcoma in Japan. *Int J Clin Oncol*. 2014;19(5):897-905. doi:10.1007/s10147-013-0627-5 |
| 36 | Hadoux J, Rey A, Duvillard P, et al. Multimodal treatment with doxorubicin, cisplatin, and ifosfamide for the treatment of advanced or metastatic uterine leiomyosarcoma: a unicentric experience. *Int J Gynecol Cancer*. 2015;25(2):296-302. doi:10.1097/IGC.0000000000000344 |
| 37 | Yamagami W, Susumu N, Ninomiya T, et al. A retrospective study on combination therapy with ifosfamide, adriamycin and cisplatin for progressive or recurrent uterine sarcoma. *Mol Clin Oncol*. 2014;2(4):591-595. doi:10.3892/mco.2014.272 |
| 38 | Pautier P, Floquet A, Chevreau C, et al. Trabectedin in combination with doxorubicin for first-line treatment of advanced uterine or soft-tissue leiomyosarcoma (LMS-02): a non-randomised, multicentre, phase 2 trial. *Lancet Oncol*. 2015;16(4):457-464. doi:10.1016/S1470-2045(15)70070-7 |
| 39 | Seddon B, Scurr M, Jones RL, et al. A phase II trial to assess the activity of gemcitabine and docetaxel as first line chemotherapy treatment in patients with unresectable leiomyosarcoma. *Clin Sarcoma Res*. 2015;5:13. doi:10.1186/s13569-015-0029-8 |
| 40 | Benson C, Ray-Coquard I, Sleijfer S, et al. Outcome of uterine sarcoma patients treated with pazopanib: A retrospective analysis based on two European Organisation for Research and Treatment of Cancer (EORTC) Soft Tissue and Bone Sarcoma Group (STBSG) clinical trials 62043 and 62072. *Gynecol Oncol*. 2016;142(1):89-94. doi:10.1016/j.ygyno.2016.03.024 |
| 41 | Hensley ML, Patel SR, von Mehren M, et al. Efficacy and safety of trabectedin or dacarbazine in patients with advanced uterine leiomyosarcoma after failure of anthracycline-based chemotherapy: Subgroup analysis of a phase 3, randomized clinical trial. *Gynecol Oncol*. 2017;146(3):531-537. doi:10.1016/j.ygyno.2017.06.018 |
| 42 | Gelderblom H, Judson IR, Benson C, et al. Treatment patterns and clinical outcomes with pazopanib in patients with advanced soft tissue sarcomas in a compassionate use setting: results of the SPIRE study. *Acta Oncol*. 2017;56(12):1769-1775. doi:10.1080/0284186X.2017.1332779 |
| 43 | Kim HJ, Kim Y, Lee SJ, Lee J, Park SH. Pazopanib monotherapy in the treatment of pretreated, metastatic uterine sarcoma: a single-center retrospective study. *J Gynecol Oncol*. 2018;29(1):e3. doi:10.3802/jgo.2018.29.e3 |
| 44 | Gadducci A, Grosso F, Scambia G, et al. A phase II randomised (calibrated design) study on the activity of the single-agent trabectedin in metastatic or locally relapsed uterine leiomyosarcoma. *Br J Cancer*. 2018;119(5):565-571. doi:10.1038/s41416-018-0190-y |
| 45 | Sunar V, Korkmaz V, Akin S, et al. Efficacy of Pazopanib in patients with metastatic uterine sarcoma: A multi-institutional study. *J BUON*. 2019;24(6):2327-2332. http://www.ncbi.nlm.nih.gov/pubmed/31983102 |
| Excluded studies | |
| 1. | Muss HB, Bundy BN, Adcock L, Beecham J. Mitoxantrone in the treatment of advanced uterine sarcoma. A phase II trial of the Gynecologic Oncology Group. *Am J Clin Oncol*. 1990;13(1):32-34. doi:10.1097/00000421-199002000-00009 |
| 2. | Thigpen T, Blessing JA, Yordan E, Valea F, Vaccarello L. Phase II trial of etoposide in leiomyosarcoma of the uterus: a Gynecologic Oncology Group study. *Gynecol Oncol*. 1996;63(1):120-122. doi:10.1006/gyno.1996.0289 |
| 3. | Mackay HJ, Buckanovich RJ, Hirte H, et al. A phase II study single agent of aflibercept (VEGF Trap) in patients with recurrent or metastatic gynecologic carcinosarcomas and uterine leiomyosarcoma. A trial of the Princess Margaret Hospital, Chicago and California Cancer Phase II Consortia. *Gynecol Oncol*. 2012;125(1):136-140. doi:10.1016/j.ygyno.2011.11.042 |
| 4. | Duska LR, Blessing JA, Rotmensch J, et al. A Phase II evaluation of ixabepilone (IND #59699, NSC #710428) in the treatment of recurrent or persistent leiomyosarcoma of the uterus: an NRG Oncology/Gynecologic Oncology Group Study. *Gynecol Oncol*. 2014;135(1):44-48. doi:10.1016/j.ygyno.2014.07.101 |
| 5. | Hyman DM, Sill MW, Lankes HA, et al. A phase 2 study of alisertib (MLN8237) in recurrent or persistent uterine leiomyosarcoma: An NRG Oncology/Gynecologic Oncology Group study 0231D. *Gynecol Oncol*. 2017;144(1):96-100. doi:10.1016/j.ygyno.2016.10.036 |

# Supplementary Table S 2: Included and excluded studies in meta-analysis and meta regression analysis of Disease Control Rate (DCR) Included studies

| 1. | Azizi F, Bitran J, Javehari G, Herbst AL. Remission of uterine leiomyosarcomas treated with vincristine, Adriamycin, and dimethyl-triazeno-imidazole carboximide. *Am J Obstet Gynecol*. 1979;133(4):379-381. doi:10.1016/0002-9378(79)90055-3 |
| --- | --- |
| 2. | Hannigan E V., Freedman RS, Elder KW, Rutledge FN. Treatment of advanced uterine sarcoma with vincristine, actinomycin D, and cyclophosphamide. *Gynecol Oncol*. 1983;15(2):224-229. doi:10.1016/0090-8258(83)90078-1 |
| 3. | Omura GA, Major FJ, Blessing JA, et al. A randomized study of adriamycin with and without dimethyl triazenoimidazole carboxamide in advanced uterine sarcomas. *Cancer*. 1983;52(4):626-632. doi:10.1002/1097-0142(19830815)52:4<626::aid-cncr2820520409>3.0.co;2-e |
| 4. | Thigpen JT, Blessing JA, Homesley HD, Hacker N, Curry SL. Phase II trial of piperazinedione in patients with advanced or recurrent uterine sarcoma. A Gynecologic Oncology Group study. *Am J Clin Oncol*. 1985;8(5):350-352. doi:10.1097/00000421-198510000-00002 |
| 5. | Thigpen JT, Blessing JA, Wilbanks GD. Cisplatin as second-line chemotherapy in the treatment of advanced or recurrent leiomyosarcoma of the uterus. A phase II trial of the Gynecologic Oncology Group. *Am J Clin Oncol*. 1986;9(1):18-20. doi:10.1097/00000421-198602000-00005 |
| 6. | Hawkins RE, Wiltshaw E, Mansi JL. Ifosfamide with and without adriamycin in advanced uterine leiomyosarcoma. *Cancer Chemother Pharmacol*. 1990;26 Suppl:S26-9. doi:10.1007/BF00685412 |
| 7. | Muss HB, Bundy BN, Adcock L, Beecham J. Mitoxantrone in the treatment of advanced uterine sarcoma. A phase II trial of the Gynecologic Oncology Group. *Am J Clin Oncol*. 1990;13(1):32-34. doi:10.1097/00000421-199002000-00009 |
| 8. | Thigpen JT, Blessing JA, Beecham J, Homesley H, Yordan E. Phase II trial of cisplatin as first-line chemotherapy in patients with advanced or recurrent uterine sarcomas: a Gynecologic Oncology Group study. *J Clin Oncol*. 1991;9(11):1962-1966. doi:10.1200/JCO.1991.9.11.1962 |
| 9. | Sutton GP, Blessing JA, Barrett RJ, McGehee R. Phase II trial of ifosfamide and mesna in leiomyosarcoma of the uterus: a Gynecologic Oncology Group study. *Am J Obstet Gynecol*. 1992;166(2):556-559. doi:10.1016/0002-9378(92)91671-v |
| 10. | Currie J, Blessing JA, Muss HB, Fowler J, Berman M, Burke TW. Combination chemotherapy with hydroxyurea, dacarbazine (DTIC), and etoposide in the treatment of uterine leiomyosarcoma: a Gynecologic Oncology Group study. *Gynecol Oncol*. 1996;61(1):27-30. doi:10.1006/gyno.1996.0091 |
| 11. | Thigpen T, Blessing JA, Yordan E, Valea F, Vaccarello L. Phase II trial of etoposide in leiomyosarcoma of the uterus: a Gynecologic Oncology Group study. *Gynecol Oncol*. 1996;63(1):120-122. doi:10.1006/gyno.1996.0289 |
| 12. | Sutton G, Blessing JA, Malfetano JH. Ifosfamide and doxorubicin in the treatment of advanced leiomyosarcomas of the uterus: a Gynecologic Oncology Group study. *Gynecol Oncol*. 1996;62(2):226-229. doi:10.1006/gyno.1996.0220 |
| 13. | Resnik E, Chambers SK, Carcangiu ML, Kohorn EI, Schwartz PE, Chambers IT. Malignant uterine smooth muscle tumors: role of etoposide, cisplatin, and doxorubicin (EPA) chemotherapy. *J Surg Oncol*. 1996;63(3):145-147. doi:10.1002/(SICI)1096-9098(199611)63:3<145::AID-JSO3>3.0.CO;2-D |
| 14. | Rose PG, Blessing JA, Soper JT, Barter JF. Prolonged oral etoposide in recurrent or advanced leiomyosarcoma of the uterus: a gynecologic oncology group study. *Gynecol Oncol*. 1998;70(2):267-271. doi:10.1006/gyno.1998.5080 |
| 15. | Sutton G, Blessing JA, Ball H. Phase II trial of paclitaxel in leiomyosarcoma of the uterus: a gynecologic oncology group study. *Gynecol Oncol*. 1999;74(3):346-349. doi:10.1006/gyno.1999.5463 |
| 16 | Miller DS, Blessing JA, Kilgore LC, Mannel R, Van Le L. Phase II trial of topotecan in patients with advanced, persistent, or recurrent uterine leiomyosarcomas: a Gynecologic Oncology Group Study. *Am J Clin Oncol*. 2000;23(4):355-357. doi:10.1097/00000421-200008000-00009 |
| 17. | Smith HO, Blessing JA, Vaccarello L. Trimetrexate in the treatment of recurrent or advanced leiomyosarcoma of the uterus: a phase II study of the Gynecologic Oncology Group. *Gynecol Oncol*. 2002;84(1):140-144. doi:10.1006/gyno.2001.6482 |
| 18. | Pearl ML, Inagami M, McCauley DL, Valea FA, Chalas E, Fischer M. Mesna, doxorubicin, ifosfamide, and dacarbazine (MAID) chemotherapy for gynecological sarcomas. *Int J Gynecol Cancer*. 2002;12(6):745-748. doi:10.1046/j.1525-1438.2002.01139.x |
| 19. | Edmonson JH, Blessing JA, Cosin JA, Miller DS, Cohn DE, Rotmensch J. Phase II study of mitomycin, doxorubicin, and cisplatin in the treatment of advanced uterine leiomyosarcoma: a Gynecologic Oncology Group study. *Gynecol Oncol*. 2002;85(3):507-510. doi:10.1006/gyno.2002.6661 |
| 20. | Hensley ML, Maki R, Venkatraman E, et al. Gemcitabine and docetaxel in patients with unresectable leiomyosarcoma: results of a phase II trial. *J Clin Oncol*. 2002;20(12):2824-2831. doi:10.1200/JCO.2002.11.050 |
| 21. | Gallup DG, Blessing JA, Andersen W, Morgan MA, Gynecologic Oncology Group Study. Evaluation of paclitaxel in previously treated leiomyosarcoma of the uterus: a gynecologic oncology group study. *Gynecol Oncol*. 2003;89(1):48-51. doi:10.1016/s0090-8258(02)00136-1 |
| 22. | Sutton G, Blessing J, Hanjani P, Kramer P, Gynecologic Oncology Group. Phase II evaluation of liposomal doxorubicin (Doxil) in recurrent or advanced leiomyosarcoma of the uterus: a Gynecologic Oncology Group study. *Gynecol Oncol*. 2005;96(3):749-752. doi:10.1016/j.ygyno.2004.11.036 |
| 23. | Long HJ, Blessing JA, Sorosky J. Phase II trial of dacarbazine, mitomycin, doxorubicin, and cisplatin with sargramostim in uterine leiomyosarcoma: a Gynecologic Oncology Group study. *Gynecol Oncol*. 2005;99(2):339-342. doi:10.1016/j.ygyno.2005.06.002 |
| 24. | D’Adamo DR, Anderson SE, Albritton K, et al. Phase II study of doxorubicin and bevacizumab for patients with metastatic soft-tissue sarcomas. *J Clin Oncol*. 2005;23(28):7135-7142. doi:10.1200/JCO.2005.16.139 |
| 25. | Boyar MS, Hesdorffer M, Keohan ML, Jin Z, Taub RN. Phase II Study of Temozolomide and Thalidomide in Patients with Unresectable or Metastatic Leiomyosarcoma. *Sarcoma*. 2008;2008:412503. doi:10.1155/2008/412503 |
| 26. | Anderson S, Aghajanian C. Temozolomide in uterine leiomyosarcomas. *Gynecol Oncol*. 2005;98(1):99-103. doi:10.1016/j.ygyno.2005.03.018 |
| 27. | Hensley ML, Blessing JA, Degeest K, Abulafia O, Rose PG, Homesley HD. Fixed-dose rate gemcitabine plus docetaxel as second-line therapy for metastatic uterine leiomyosarcoma: a Gynecologic Oncology Group phase II study. *Gynecol Oncol*. 2008;109(3):323-328. doi:10.1016/j.ygyno.2008.02.024 |
| 28. | Hensley ML, Blessing JA, Mannel R, Rose PG. Fixed-dose rate gemcitabine plus docetaxel as first-line therapy for metastatic uterine leiomyosarcoma: a Gynecologic Oncology Group phase II trial. *Gynecol Oncol*. 2008;109(3):329-334. doi:10.1016/j.ygyno.2008.03.010 |
| 29. | Hensley ML, Sill MW, Scribner DR, et al. Sunitinib malate in the treatment of recurrent or persistent uterine leiomyosarcoma: a Gynecologic Oncology Group phase II study. *Gynecol Oncol*. 2009;115(3):460-465. doi:10.1016/j.ygyno.2009.09.011 |
| 30. | Maki RG, D’Adamo DR, Keohan ML, et al. Phase II study of sorafenib in patients with metastatic or recurrent sarcomas. *J Clin Oncol*. 2009;27(19):3133-3140. doi:10.1200/JCO.2008.20.4495 |
| 31. | Sleijfer S, Ray-Coquard I, Papai Z, et al. Pazopanib, a multikinase angiogenesis inhibitor, in patients with relapsed or refractory advanced soft tissue sarcoma: a phase II study from the European organisation for research and treatment of cancer-soft tissue and bone sarcoma group (EORTC study 620. *J Clin Oncol*. 2009;27(19):3126-3132. doi:10.1200/JCO.2008.21.3223 |
| 32. | Judson IR, Blay J, Chawla SP, et al. Trabectedin (Tr) in the treatment of advanced uterine leiomyosarcomas (U-LMS): Results of a pooled analysis of five single-agent phase II studies using the recommended dose. *J Clin Oncol*. 2010;28(15_suppl):10028-10028. doi:10.1200/jco.2010.28.15_suppl.10028 |
| 33. | Sanfilippo R, Grosso F, Jones RL, et al. Trabectedin in advanced uterine leiomyosarcomas: a retrospective case series analysis from two reference centers. *Gynecol Oncol*. 2011;123(3):553-556. doi:10.1016/j.ygyno.2011.08.016 |
| 34. | Monk BJ, Blessing JA, Street DG, Muller CY, Burke JJ, Hensley ML. A phase II evaluation of trabectedin in the treatment of advanced, persistent, or recurrent uterine leiomyosarcoma: a gynecologic oncology group study. *Gynecol Oncol*. 2012;124(1):48-52. doi:10.1016/j.ygyno.2011.09.019 |
| 35. | Mackay HJ, Buckanovich RJ, Hirte H, et al. A phase II study single agent of aflibercept (VEGF Trap) in patients with recurrent or metastatic gynecologic carcinosarcomas and uterine leiomyosarcoma. A trial of the Princess Margaret Hospital, Chicago and California Cancer Phase II Consortia. *Gynecol Oncol*. 2012;125(1):136-140. doi:10.1016/j.ygyno.2011.11.042 |
| 36. | Yoo HJ, Lim MC, Lim S, et al. Phase II study of paclitaxel in combination with carboplatin for patients with recurrent or persistent uterine sarcoma. *Arch Gynecol Obstet*. 2012;286(6):1529-1535. doi:10.1007/s00404-012-2466-4 |
| 37. | Pautier P, Floquet A, Penel N, et al. Randomized multicenter and stratified phase II study of gemcitabine alone versus gemcitabine and docetaxel in patients with metastatic or relapsed leiomyosarcomas: a Federation Nationale des Centres de Lutte Contre le Cancer (FNCLCC) French Sarcoma Group. *Oncologist*. 2012;17(9):1213-1220. doi:10.1634/theoncologist.2011-0467 |
| 38. | Takano T, Niikura H, Ito K, et al. Feasibility study of gemcitabine plus docetaxel in advanced or recurrent uterine leiomyosarcoma and undifferentiated endometrial sarcoma in Japan. *Int J Clin Oncol*. 2014;19(5):897-905. doi:10.1007/s10147-013-0627-5 |
| 39. | Hadoux J, Rey A, Duvillard P, et al. Multimodal treatment with doxorubicin, cisplatin, and ifosfamide for the treatment of advanced or metastatic uterine leiomyosarcoma: a unicentric experience. *Int J Gynecol Cancer*. 2015;25(2):296-302. doi:10.1097/IGC.0000000000000344 |
| 40. | Yamagami W, Susumu N, Ninomiya T, et al. A retrospective study on combination therapy with ifosfamide, adriamycin and cisplatin for progressive or recurrent uterine sarcoma. *Mol Clin Oncol*. 2014;2(4):591-595. doi:10.3892/mco.2014.272 |
| 41. | Duska LR, Blessing JA, Rotmensch J, et al. A Phase II evaluation of ixabepilone (IND #59699, NSC #710428) in the treatment of recurrent or persistent leiomyosarcoma of the uterus: an NRG Oncology/Gynecologic Oncology Group Study. *Gynecol Oncol*. 2014;135(1):44-48. doi:10.1016/j.ygyno.2014.07.101 |
| 42. | Pautier P, Floquet A, Chevreau C, et al. Trabectedin in combination with doxorubicin for first-line treatment of advanced uterine or soft-tissue leiomyosarcoma (LMS-02): a non-randomised, multicentre, phase 2 trial. *Lancet Oncol*. 2015;16(4):457-464. doi:10.1016/S1470-2045(15)70070-7 |
| 43. | Seddon B, Scurr M, Jones RL, et al. A phase II trial to assess the activity of gemcitabine and docetaxel as first line chemotherapy treatment in patients with unresectable leiomyosarcoma. *Clin Sarcoma Res*. 2015;5:13. doi:10.1186/s13569-015-0029-8 |
| 44. | Benson C, Ray-Coquard I, Sleijfer S, et al. Outcome of uterine sarcoma patients treated with pazopanib: A retrospective analysis based on two European Organisation for Research and Treatment of Cancer (EORTC) Soft Tissue and Bone Sarcoma Group (STBSG) clinical trials 62043 and 62072. *Gynecol Oncol*. 2016;142(1):89-94. doi:10.1016/j.ygyno.2016.03.024 |
| 45. | Hensley ML, Patel SR, von Mehren M, et al. Efficacy and safety of trabectedin or dacarbazine in patients with advanced uterine leiomyosarcoma after failure of anthracycline-based chemotherapy: Subgroup analysis of a phase 3, randomized clinical trial. *Gynecol Oncol*. 2017;146(3):531-537. doi:10.1016/j.ygyno.2017.06.018 |
| 46. | Gelderblom H, Judson IR, Benson C, et al. Treatment patterns and clinical outcomes with pazopanib in patients with advanced soft tissue sarcomas in a compassionate use setting: results of the SPIRE study. *Acta Oncol*. 2017;56(12):1769-1775. doi:10.1080/0284186X.2017.1332779 |
| 47. | Hyman DM, Sill MW, Lankes HA, et al. A phase 2 study of alisertib (MLN8237) in recurrent or persistent uterine leiomyosarcoma: An NRG Oncology/Gynecologic Oncology Group study 0231D. *Gynecol Oncol*. 2017;144(1):96-100. doi:10.1016/j.ygyno.2016.10.036 |
| 48. | Kim HJ, Kim Y, Lee SJ, Lee J, Park SH. Pazopanib monotherapy in the treatment of pretreated, metastatic uterine sarcoma: a single-center retrospective study. *J Gynecol Oncol*. 2018;29(1):e3. doi:10.3802/jgo.2018.29.e3 |
| 49. | Gadducci A, Grosso F, Scambia G, et al. A phase II randomised (calibrated design) study on the activity of the single-agent trabectedin in metastatic or locally relapsed uterine leiomyosarcoma. *Br J Cancer*. 2018;119(5):565-571. doi:10.1038/s41416-018-0190-y |
| 50. | Sunar V, Korkmaz V, Akin S, et al. Efficacy of Pazopanib in patients with metastatic uterine sarcoma: A multi-institutional study. *J BUON*. 2019;24(6):2327-2332. http://www.ncbi.nlm.nih.gov/pubmed/31983102 |
| **Excluded studies** | |
|  | |
| 1. | Hannigan E V., Freedman RS, Elder KW, Rutledge FN. Treatment of advanced uterine sarcoma with vincristine, actinomycin D, and cyclophosphamide. *Gynecol Oncol*. 1983;15(2):224-229. doi:10.1016/0090-8258(83)90078-1 |
| 2. | Rose PG, Blessing JA, Soper JT, Barter JF. Prolonged oral etoposide in recurrent or advanced leiomyosarcoma of the uterus: a gynecologic oncology group study. *Gynecol Oncol*. 1998;70(2):267-271. doi:10.1006/gyno.1998.5080 |
| 3. | Sleijfer S, Ray-Coquard I, Papai Z, et al. Pazopanib, a multikinase angiogenesis inhibitor, in patients with relapsed or refractory advanced soft tissue sarcoma: a phase II study from the European organisation for research and treatment of cancer-soft tissue and bone sarcoma group (EORTC study 620. *J Clin Oncol*. 2009;27(19):3126-3132. doi:10.1200/JCO.2008.21.3223 |

**Supplementary Table S3.** Basic characteristics of the studies included in sub-meta-analysis.

| Author (Year Ref.) | Patient’s Status | Prior systemic therapy (n) | Prior radiation therapy (n) | Prior surgical treatment (n) | Organs Involved in Metastasis | Disease evaluation modalities |
| --- | --- | --- | --- | --- | --- | --- |
| Azizi F ,1979 (Azizi et al., 1979) | M | 0 | 0 | 6 | Lung, Liver, Pelvis, Vagina | CXR |
| Hannigan E, 1983 (Hannigan et al., 1983) | M/R/Ad | NR | NA | NA | Abdomen, Pelvis | Radiographic |
| Omura G, 1983 (Omura et al., 1983)^a^ | R/Pr | 3 | 13 | NA | Lung | Radiographic |
| Omura G, 1983 (Omura et al., 1983)^b^ | R/Pr | 2 | 9 | NA | Lung | Radiographic |
| Thigpen J, 1985 (Thigpen et al., 1985) | Ad/R | 11 | 8 | 11 | Extrapelvic | Radiographic |
| Thigpen J, 1986 (Thigpen et al., 1986) | Ad/R | 19 | 4 | 18 | Extrapelvic | Radiographic |
| Hawkins R, 1990 (Hawkins et al., 1990)^a^ | M/LA | 2 | 0 | 10 | Lung, Liver Bone | CXR, CT |
| Hawkins R, 1990 (Hawkins et al., 1990)^b^ | M/LA | 4 | 2 | 8 | Lung, Liver Bone | CXR, CT |
| Muss H, 1990 (Muss et al., 1990) | Ad | 8 | 4 | 10 | Pelvic, Extrapelvic | CXR, Radiographic |
| Thigpen J, 1991 (Thigpen et al., 1991) | Ad/R | 2 | 8 | 30 | Pelvic, Extrapelvic | Radiographic |
| Sutton G, 1992 (Sutton et al., 1992) | Ad/R | NR | 15 | 26 | NR | CT |
| Currie J, 1996 (Currie et al., 1996) | Ad/R | 6 | 11 | 35 | Chest, Abdomen, Pelvic | CT, MRI, CXR,USG |
| Thigpen T, 1996 (Thigpen et al., 1996) | R/P | 0 | 7 | NR | Pelvic, Extrapelvic | NA |
| Sutton G, 1996 (Sutton et al., 1996) | Ad/R | NR | 9 | 29 | Abdomen, Pelvis | CT, CXR |
| Resnik E, 1996 (Resnik et al., 1996) | Ad/R | NR | NR | 7 | Lung | NA |
| Rose P, 1998 (Rose et al., 1998) | Ad/R | 27 | 6 | NR | Pelvic, Extrapelvic | Radiographic |
| Sutton G, 1999 (Sutton et al., 1999) | M/Ad | NR | 8 | 33 | LA, Extrapelvic | CT, CXR |
| Miller D, 2000 (Miller et al., 2000) | Ad/P/R | 0 | 8 | 33 | Extrapelvic | Radiographic |
| Smith H, 2002 (Smith et al., 2002) | Ad/R | 10 | 7 | NR | LA, Extrapelvic | NA |
| Pearl M, 2002 (Pearl et al., 2002) | Ad/R | NR | NR | 5 | NA | NA |
| Edmonson J, 2002 (Edmonson et al., 2002) | Ad | 0 | 8 | NR | Extrapelvic | CT, MRI |
| Hensley M, 2002 (Hensley et al., 2002) | M/Un | 16 | 14 | NR | NA | CT |
| Gallup D, 2003 (Gallup et al., 2003) | Ad/R | 39 | 15 | NR | Lung, Liver | Radiographic |
| Look K, 2004 (Look et al., 2004) | R/P | 35 | 11 | NR | NR | CT |
| Sutton G, 2005 (Sutton et al., 2005) | Ad/R | 0 | 11 | NR | NA | CT, CXR |
| Long III H, 2005 (Long III et al., 2005) | Ad/P/R | 0 | 7 | 16 | NA | Radiographic |
| D'Adamo D, 2005 (D'Adamo et al., 2005) | M | 6 | 2 | 7 | Lung, Liver | CT, CXR |
| Boyar M, 2005 (Boyar et al., 2005) | M/Un | 10 | NA | NA | Lung, Liver, Bone | CT |
| Anderson S, 2005 (Anderson and Aghajanian, 2005) | M/R | 12 | 4 | NR | Lung, Bone, pelvic | CT |
| Hensley M, 2008 (Hensley et al., 2008a) | Ad/R | 45 | 17 | NR | NA | CT |
| Hensley M, 2008 (Hensley et al., 2008b) | Ad/Un | 0 | 12 | 0 | NA | CT/ Pelvic exam |
| Hensley M, 2009 (Hensley et al., 2009) | R/P | 23 | 9 | NR | NR | CT, MRI |
| Maki R, 2009 (Maki et al., 2009) | M/R | 37 | NR | NR | Lung | Radiographic |
| Sleijfer S, 2009 (Sleijfer et al., 2009b) | P/R | NA | NR | NR | NA | CT, MRI |
| Judson I, 2010 (Judson et al., 2010) | Ad | 56 | 30 | 61 | NA | CT |
| Sanfilippo R, 2011 (Sanfilippo et al., 2011) | Ad | 66 | NR | 64 | Lung, Liver, Bone | CT, MRI |
| Monk B, 2012 (Monk et al., 2012) | Ad/P/R | 0 | 7 | NR | NR | Radiographic |
| Mackay H, 2012 (Mackay et al., 2012) | M/R | 27 | 14 | NR | Lung, Liver, Pelvis, Nodes | CT, MRI |
| Yoo H, 2012 (Yoo et al., 2012) | R/Pr | 2 | 6 | 8 | NA | CT |
| Pautier P, 2012 (Pautier et al., 2012)^a^ | M/Un | 21 | 12 | NR | Lung, Liver | CT |
| Pautier P, 2012 (Pautier et al., 2012)^b^ | M/Un | 18 | 16 | NR | Lung, Liver | CT |
| Takano T, 2014 (Takano et al., 2014) | Ad/R/Un | 5 | NA | 10 | Lung, Liver, Pelvis, Omentum | CT |
| Hadoux J, 2014 (2015) | M/Ad | 0 | 8 | 31 | Lung, Liver, Pelvis, Bone | CT, MRI |
| Yamagami W, 2014 (Yamagami et al., 2014) | R/Pr | 0 | NR | 6 | NA | CT |
| Duska L, 2014 (Duska et al., 2014) | R/P | 21 | 6 | NR | NA | CT |
| Pautier P, 2015 (Pautier et al., 2015) | M/Un | NR | 17 | 14 | Lung, Liver, Bone | CT, MRI |
| Seddon B, 2015 (Seddon et al., 2015) | Ad/R | NR | 9 | 10 | Lung, Liver | CT |
| Benson C, 2016 (Benson et al., 2016) | M | 27 T: 3 | 11 | 9 | Lung, Liver, Bone | Radiographic |
| Hensley M, 2017 (Hensley et al., 2017)^a^ | M/Un | 88 | 32 | 85 | NR | NR |
| Hensley M, 2017 (Hensley et al., 2017)^b^ | M/Un | 143 | 70 | 140 | NR | NR |
| Gelderblom H, 2017 (Gelderblom et al., 2017) | Ad | NR | NR | NR | Lung | Radiographic |
| Hyman D, 2017 (Hyman et al., 2017) | R/P | 21, Im: 2 | 21 | 4 | NR | CT, MRI |
| Kim H, 2018 (Kim et al., 2018) | M | 17 | 14 | 27 | Lung, Liver, Bone | CT, CXR |
| Gadducci A, 2018 (Gadducci et al., 2018)^a^ | P/R/Pr | NA/84 | NA/14 | NA/115 | Lung, Liver, Bone , Spleen, Peritoneum | Radiographic |
| Gadducci A, 2018 (Gadducci et al., 2018)^b^ |  |  |  |  |  |  |
| Sunar V, 2019 (Sunar et al., 2019a) | M | 28 | NR | 27 | Lung, Liver, Bone, Pelvis | CT, MRI |
| Abbreviations : Ad, Advanced; Ad/R, Advanced/ Recurrent; Ad/P/R, Advanced/Persistent/ Recurrent; Ad/R/Un, Adanced/ Recurrent/ Unresectable; LA, Locally Advanced; M, Metastatic; M/R/Ad, Metastatic/ Recurrent/ Advanced; M/LA, Metastatic/ Locally Advanced; M/Un, Metastatic/ Unresecatble; M/R,Metastatic/Recurrent; Pr, Progressive; P, Persistent; P/R/Pr, Persistent / Recurrent/Progressive; R, Recurrent; R/Pr, Recurrent/ Progressive; R/P, Recurrent/ Persistent , Un, Unresectable; Rx, Therapy; NR, Not reported; NA, Not available; Abd, Abdomen; ; CT, Computed tomography; MRI, Magnetic resonance immaging; CXR, Chest X ray; USG, ultrasonography; NOS, New Casttle Ottawa Scale; | | | | | | |

**Supplementary Table S4.** Quality assessment of included studies according to the Newcastle-Ottawa Scale (NOS).

| **Author (Year)** | **Analyzing** | Case | | Control | | Comparability | | \Exposure | | | | NOS |
| --- | --- | --- | --- | --- | --- | --- | --- | --- | --- | --- | --- | --- |
|  |  | Definition | Representativeness | Selection | Definition | Important factors | Other factors | Secure record | Blind | Method | Non-response rate |  |
| Azizi F (1979) | ANT + ALK + Vinca | ★ | ★ | ☆ | ☆ | ★ | ☆ | ★ | ☆ | ★ | ★ | 6 |
| Hannigan E (1983) | ANT + ALK + Vinca | ★ | ★ | ☆ | ☆ | ★ | ★ | ★ | ☆ | ★ | ★ | 7 |
| Omura G (1983) | ANT + ANT + ALK | ★ | ★ | ☆ | ★ | ★ | ★ | ★ | ☆ | ★ | ★ | 8 |
| Thigpen J (1985) | Top II alone | ★ | ★ | ☆ | ☆ | ★ | ★ | ★ | ☆ | ★ | ★ | 7 |
| Thigpen J (1986) | ALK alone | ★ | ★ | ☆ | ☆ | ★ | ☆ | ★ | ☆ | ★ | ★ | 6 |
| Hawkins R (1990) | ANT + ALK + ALK | ★ | ★ | ☆ | ★ | ★ | ☆ | ★ | ☆ | ★ | ★ | 7 |
| Muss H (1990) | ANT alone | ★ | ★ | ☆ | ☆ | ★ | ★ | ★ | ☆ | ★ | ★ | 7 |
| Thigpen J (1991) | ALK alone | ★ | ★ | ☆ | ☆ | ★ | ☆ | ★ | ☆ | ★ | ★ | 6 |
| Sutton G (1992) | ALK alone | ★ | ★ | ☆ | ☆ | ★ | ★ | ★ | ☆ | ★ | ★ | 7 |
| Currie J (1996) | Antimetabolite +ALK +TopII | ★ | ★ | ☆ | ☆ | ★ | ★ | ★ | ☆ | ★ | ★ | 7 |
| Thigpen T (1996) | Top II alone | ★ | ★ | ☆ | ☆ | ★ | ★ | ★ | ☆ | ★ | ★ | 7 |
| Sutton G (1996) | ANT + ALK | ★ | ★ | ☆ | ☆ | ★ | ★ | ★ | ☆ | ★ | ★ | 7 |
| Resnik E (1996) | ANT+ ALK+ TopII | ★ | ★ | ☆ | ☆ | ★ | ★ | ★ | ☆ | ★ | ★ | 7 |
| Rose P (1998) | Top II alone | ★ | ★ | ☆ | ☆ | ★ | ★ | ★ | ☆ | ★ | ★ | 7 |
| Sutton G (1999) | Taxane alone | ★ | ★ | ☆ | ☆ | ★ | ★ | ★ | ☆ | ★ | ★ | 7 |
| Miller D (2000) | Top II alone | ★ | ★ | ☆ | ☆ | ★ | ☆ | ★ | ☆ | ★ | ★ | 6 |
| Smith H (2002) | Antimetabolite Alone | ★ | ★ | ☆ | ☆ | ★ | ☆ | ★ | ☆ | ★ | ★ | 7 |
| Pearl M (2002) | ANT + ALK | ★ | ★ | ☆ | ☆ | ★ | ★ | ★ | ☆ | ★ | ★ | 7 |
| Edmonson J (2002) | ANT + ALK | ★ | ★ | ☆ | ☆ | ★ | ★ | ★ | ☆ | ★ | ★ | 7 |
| Hensley M (2002) | Antimetabolite +Taxane | ★ | ★ | ☆ | ☆ | ★ | ★ | ★ | ☆ | ★ | ★ | 7 |
| Gallup D (2003) | Taxane alone | ★ | ★ | ☆ | ☆ | ★ | ★ | ★ | ☆ | ★ | ★ | 7 |
| Look K (2004) | Antimetabolite Alone | ★ | ★ | ☆ | ☆ | ★ | ★ | ★ | ☆ | ★ | ★ | 7 |
| Sutton G (2005) | ANT alone | ★ | ★ | ☆ | ☆ | ★ | ☆ | ★ | ☆ | ★ | ★ | 6 |
| Long III H (2005) | ANT + ALK | ★ | ★ | ☆ | ☆ | ★ | ★ | ★ | ☆ | ★ | ★ | 7 |
| D'Adamo D (2005) | ANT + MAB | ★ | ★ | ☆ | ☆ | ★ | ★ | ★ | ☆ | ★ | ★ | 7 |
| Boyar M (2005) | ALK + immunomodulator | ★ | ★ | ☆ | ☆ | ★ | ★ | ★ | ☆ | ★ | ★ | 7 |
| Anderson S (2005) | ALK alone | ★ | ★ | ☆ | ☆ | ★ | ☆ | ★ | ☆ | ★ | ★ | 6 |
| Hensley M (2008) | Antimetabolite + Taxane | ★ | ★ | ☆ | ☆ | ★ | ★ | ★ | ☆ | ★ | ★ | 7 |
| Hensley M (2008) | Antimetabolite + Taxane | ★ | ★ | ☆ | ☆ | ★ | ★ | ★ | ☆ | ★ | ★ | 7 |
| Hensley M (2009) | PKII alone | ★ | ★ | ☆ | ☆ | ★ | ★ | ★ | ☆ | ★ | ★ | 7 |
| Maki R (2009) | PKII alone | ★ | ★ | ☆ | ☆ | ★ | ★ | ★ | ☆ | ★ | ★ | 7 |
| Sleijfer S (2009) | PKII alone | ★ | ★ | ☆ | ☆ | ★ | ★ | ★ | ★ | ★ | ★ | 8 |
| Judson I (2010) | ALK alone | ★ | ★ | ☆ | ☆ | ★ | ★ | ★ | ☆ | ★ | ★ | 7 |
| Sanfilippo R (2011) | ALK alone | ★ | ★ | ☆ | ☆ | ★ | ★ | ★ | ☆ | ★ | ★ | 7 |
| Monk B (2012) | ALK alone | ★ | ★ | ☆ | ☆ | ★ | ★ | ★ | ☆ | ★ | ★ | 7 |
| Mackay H (2012) | ANT + MAB | ★ | ★ | ☆ | ☆ | ★ | ★ | ★ | ☆ | ★ | ★ | 7 |
| Yoo H (2012) | Antimetabolite + Taxane | ★ | ★ | ☆ | ☆ | ★ | ★ | ★ | ☆ | ★ | ★ | 6 |
| Pautier P (2012) | Antimetabolite + Taxane | ★ | ★ | ☆ | ★ | ★ | ★ | ★ | ☆ | ★ | ★ | 8 |
| Takano T (2014) | Antimetabolite + Taxane | ★ | ★ | ☆ | ☆ | ★ | ★ | ★ | ☆ | ★ | ★ | 7 |
| Hadoux J (2014) | ANT + ALK | ★ | ★ | ☆ | ☆ | ★ | ★ | ★ | ☆ | ★ | ★ | 7 |
| Yamagami W (2014) | ANT + ALK | ★ | ★ | ☆ | ☆ | ★ | ★ | ★ | ☆ | ★ | ★ | 7 |
| Duska L (2014) | ANT + MAB | ★ | ★ | ☆ | ☆ | ★ | ★ | ★ | ☆ | ★ | ★ | 7 |
| Pautier P (2015) | ANT + ALK | ★ | ★ | ☆ | ☆ | ★ | ★ | ★ | ☆ | ★ | ★ | 7 |
| Seddon B (2015) | Antimetabolite + Taxane | ★ | ★ | ☆ | ☆ | ★ | ★ | ★ | ☆ | ★ | ★ | 7 |
| Benson C (2016) | PKII alone | ★ | ★ | ☆ | ☆ | ★ | ★ | ★ | ★ | ★ | ★ | 8 |
| Hensley M (2017) | ALK agent | ★ | ★ | ☆ | ★ | ★ | ★ | ★ | ★ | ☆ | ★ | 8 |
| Gelderblom H (2017) | PKII alone | ★ | ★ | ☆ | ☆ | ★ | ★ | ★ | ☆ | ★ | ★ | 7 |
| Hyman D (2017) | PKII alone | ★ | ★ | ☆ | ☆ | ★ | ★ | ★ | ☆ | ★ | ★ | 7 |
| Kim H (2018) | PKII alone | ★ | ★ | ☆ | ☆ | ★ | ★ | ★ | ☆ | ★ | ★ | 7 |
| Gadducci A (2018) | Antimetabolite+ Taxane+ ALK | ★ | ★ | ☆ | ★ | ★ | ★ | ★ | ☆ | ★ | ★ | 8 |
| Sunar V (2019) | PKII alone | ★ | ★ | ☆ | ☆ | ★ | ★ | ★ | ★ | ★ | ★ | 8 |

**Supplementary Figure S1. .** Graphic representation of the risk of bias. The overall risk of bias was considered to be low in all eligible studies, according to the QUADAS-2 assessment. The reviewers' decisions about each risk of bias (A) and applicability concerns graph (B) presented as percentages across selected studies.

**
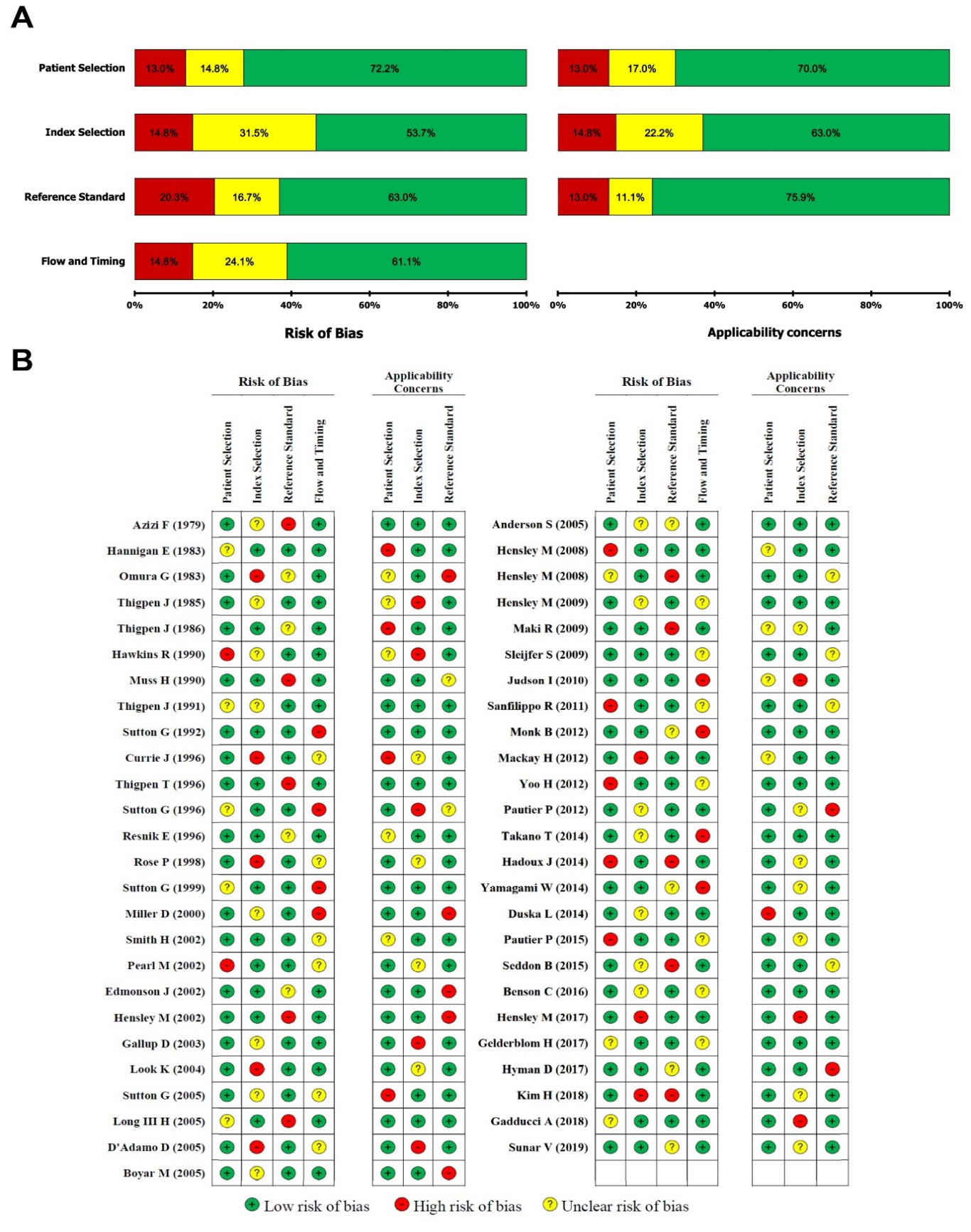
**

**Supplementary Figure S2.** Forest plots of the overall pooled objective response rates (ORRs) and disease control rates (DCRs) of advanced uterine leiomyosarcoma patients showing heterogeneity. (A) Overall pooled ORR for monotherapy (B) Overall pooled ORR of combination therapy. Heterogeneity is the variability between the study-specific effects that a random variation cannot explain. CI: confidence interval.

**
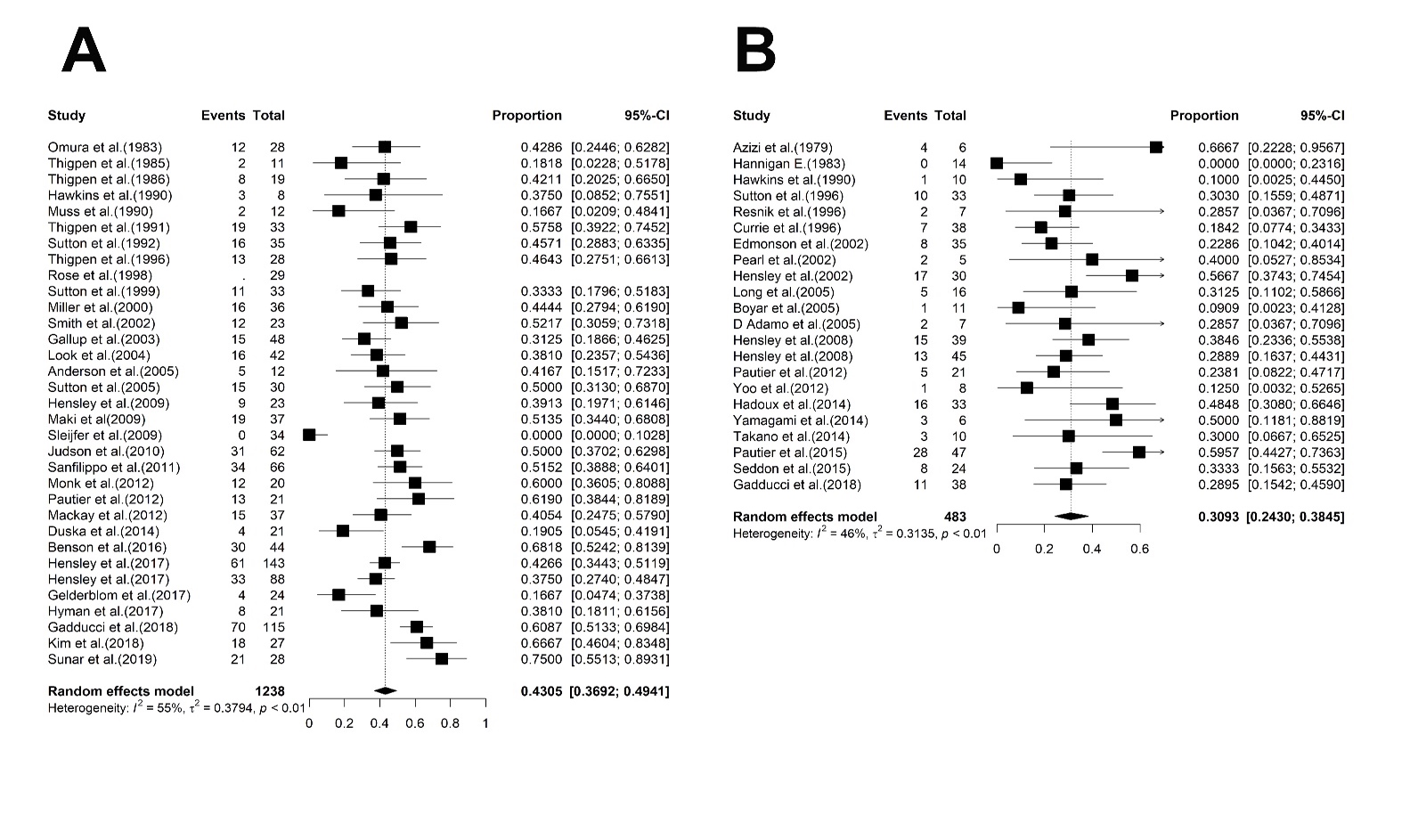
**

**Supplementary Figure S3.** Forest plots of the objective response rates (ORRs) of advanced uterine leiomyosarcoma (uLMS) showing heterogeneity. (A). pooled ORRs for the phase II and retrospective study designs. (B) pooled ORRs for the combination chemotherapy. (C) pooled ORRs for the lines of therapy. (D) pooled ORRs for the advanced FIGO stages of uterine leiomyosarcoma. Heterogeneity is the variability between the study-specific effects that a random variation cannot explain. CI: confidence interval.

**
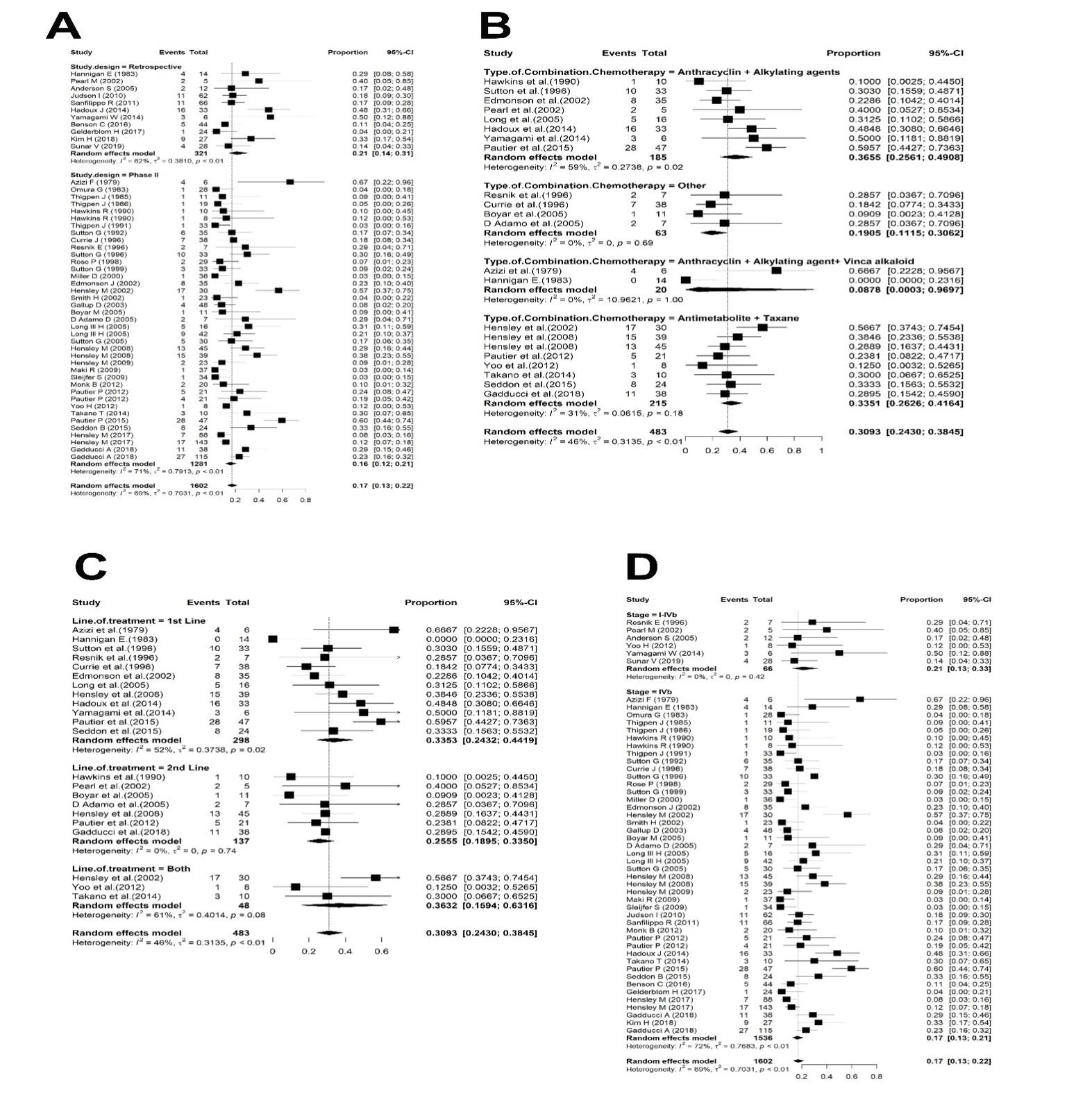
**

**Supplementary Figure S4.** Forest plots of the disease control rates (DCRs) of advanced uterine leiomyosarcoma (uLMS) showing heterogeneity. (A). pooled DCRs for the phase II and retrospective study designs. (B) pooled DCRs for the combination chemotherapy. (C) overall pooled DCRs for the monotherapies. (D, E) pooled DCRs for the lines of therapy (E) pooled ORRs for the advanced FIGO stages of uterine leiomyosarcoma. Heterogeneity is the variability between the study-specific effects that a random variation cannot explain. CI: confidence interval.

**
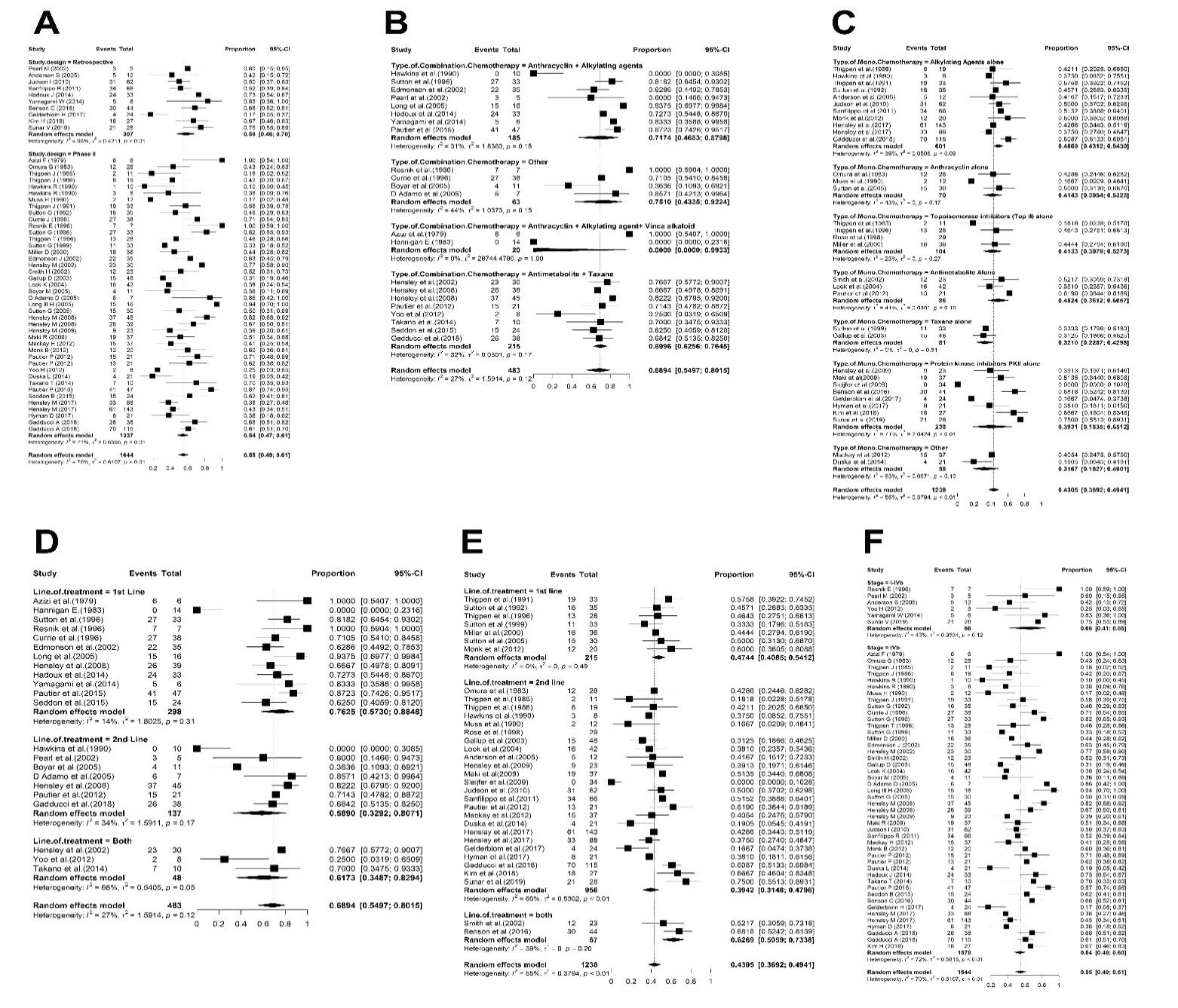
**

**Supplementary Figure S5.** Meta-regression plot to evaluate the effect on the objective response rate (ORR) for median age and publication year (A, B) and disease control rate (DCR) for median age and publication year (C, D) of advanced uLMS patients. The size of each square is proportional to the weighted percentage of each study to the standardized mean difference (SDM) ratio. Weights are retrieved from random effects analysis.


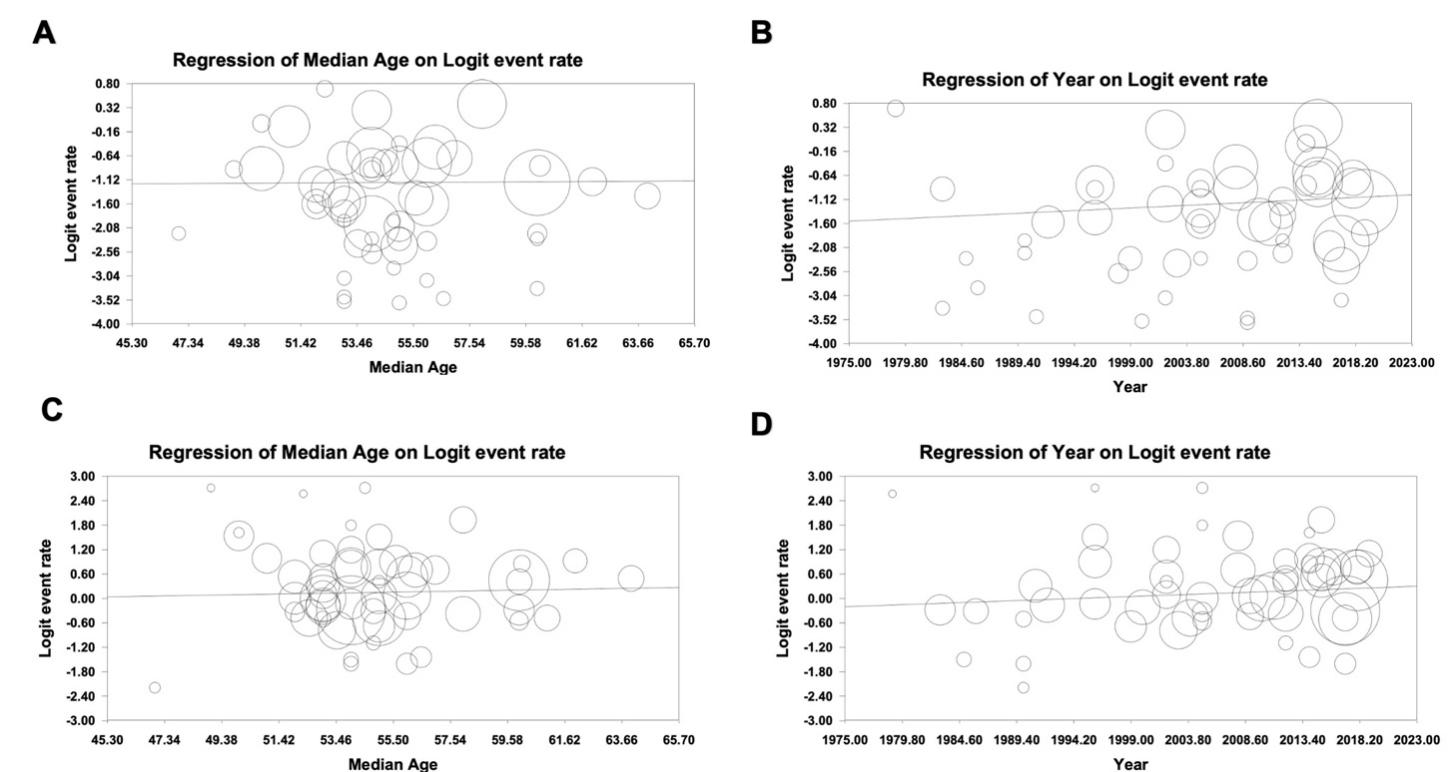


**Supplementary Figure S6.** Begg’s funnel plots and Egger’s linear regression test to evaluate the publication bias (A) funnel plot of the DCR and ORR outcome based on the regression results (B, C) forest plot for the ORR emission propagation bias and to estimate the number of missing studies.


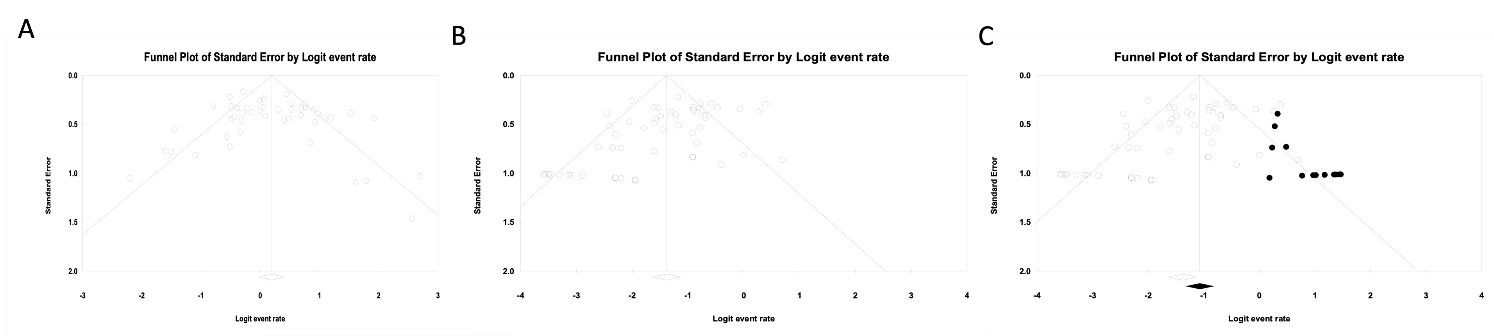

Supplement: Supplementary file 1 — Data S1: Supporting information [file CAM4-12-13894-s001.docx]
